# Using sound pulses to solve the crystal-harvesting bottleneck

**DOI:** 10.1107/S2059798318011506

**Published:** 2018-10-02

**Authors:** Yasmin N. Samara, Haley M. Brennan, Liam McCarthy, Mary T. Bollard, Denise Laspina, Jakub M. Wlodek, Stefanie L. Campos, Ramya Natarajan, Kazimierz Gofron, Sean McSweeney, Alexei S. Soares, Ludmila Leroy

**Affiliations:** a Office of Educational Programs, Brookhaven National Laboratory, Upton, NY 11973-5000, USA; b Universidade Federal de Santa Maria, 97105-900 Santa Maria-RS, Brazil; c Department of Biology, College of William and Mary, Williamsburg, VA 23187, USA; d Department of Biology, Stony Brook University, New York, NY 11794-5215, USA; e Department of Biology, York College of Pennsylvania, York, PA 17403, USA; f Department of Computer Science, Stony Brook University, New York, NY 11794-5215, USA; g Department of Clinical Nutrition, Stony Brook University, New York, NY 11794-5215, USA; h Department of Biomedical Engineering, Georgia Institute of Technology, Atlanta, GA 30332, USA; i Energy Sciences Directorate, NSLS II, Brookhaven National Laboratory, Upton, NY 11973-5000, USA; j Universidade Federal de Minas Gerais, 31270-901 Belo Horizonte-MG, Brazil

**Keywords:** crystal harvesting, acoustic droplet ejection, crystal mounting, automation, crystallography, microcrystals, high-throughput screening, drug discovery

## Abstract

Crystal harvesting has proven to be difficult to automate and remains the rate-limiting step for many structure-determination and high-throughput screening projects. This has resulted in crystals being prepared more rapidly than they can be harvested for X-ray data collection. Fourth-generation synchrotrons will support extraordinarily rapid rates of data acquisition, putting further pressure on the crystal-harvesting bottleneck. Here, a simple solution is reported in which crystals can be acoustically harvested from slightly modified MiTeGen *In Situ*-1 crystallization plates. This technique uses an acoustic pulse to eject each crystal out of its crystallization well, through a short air column and onto a micro-mesh (improving on previous work, which required separately grown crystals to be transferred before harvesting). Crystals can be individually harvested or can be serially combined with a chemical library such as a fragment library.

## Abstract 2

A simple method for using sound pulses to harvest protein crystals from a commercially available crystallization plate is described. Crystals can be grown using conventional vapor-diffusion methods and then individually harvested or serially combined with a chemical library such as a fragment library.

## 1. Introduction   

**Figure 1:**
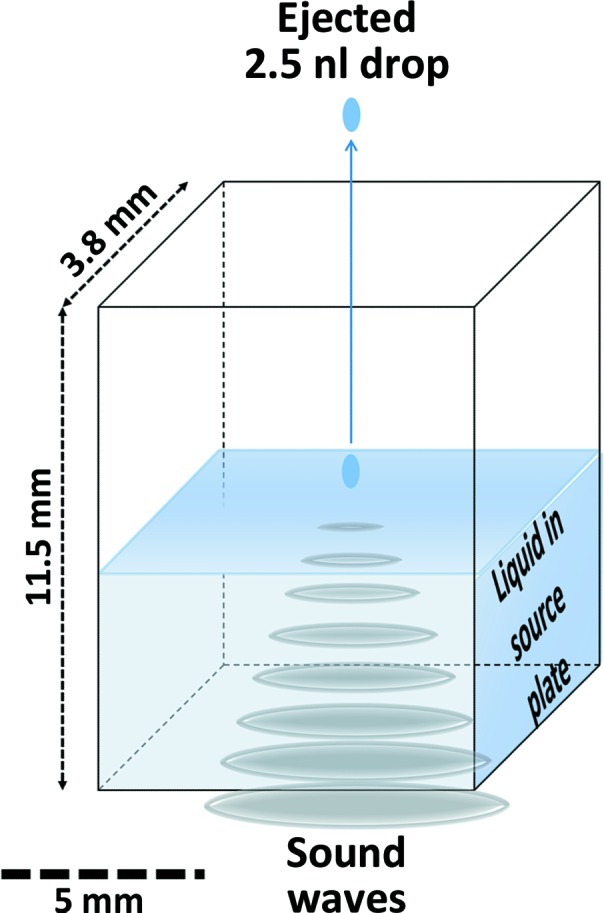
Acoustic droplet ejection (ADE). ADE uses sound energy to transfer variable micro-droplets (*e.g.* nanolitres or picolitres) of solution (including suspended solids) from a crystallization well, through a short air column (∼1 cm) to data-collection media. Sound-wave energy from the transducer is channelled to the focal point (*i.e.* the ejection zone), displacing the surface, where a controlled ejection occurs. Droplet size is governed by the wavelength of the sound emitted and this proportionality yields accurate ejected volumes. In this work, an Echo 550 liquid handler was used to harvest protein crystals from two kinds of *in situ* plates (MiTeGen *In Situ*-1) onto MiTeGen MicroMeshes.

Acoustic droplet ejection (ADE) is an automated, keyboard-driven technology that can be used for growing protein crystals (Wu *et al.*, 2016 ▸), improving the quality of protein crystals (Villaseñor *et al.*, 2010 ▸) and transferring protein crystals onto data-collection media (Soares *et al.*, 2011 ▸) such as MiTeGen MicroMesh sample holders (hereafter referred to as ‘micro-meshes’). ADE can also be used to screen chemical libraries (Collins *et al.*, 2017 ▸) using either cryocooled crystals (Yin *et al.*, 2014 ▸) or room-temperature crystals (Teplitsky *et al.*, 2015 ▸). All of these methods use momentum from a sound pulse to move liquids and/or suspended crystals from the source location through a short air column to the destination with high precision (Fig. 1 ▸). Acoustic crystal transfer using commercially available acoustic liquid handlers is gentle (no hand tools are required) and fast (2.33 ± 0.04 harvests per second; Cuttitta *et al.*, 2015 ▸). The equipment is simple to use and does not require a high level of training or manual dexterity.^1^

Commercial ADE equipment achieves high-volume transfer accuracy [±5%(*v*/*v*); Sackmann *et al.*, 2016 ▸] and precise droplet trajectory (±20 µm) in part by using specialized labware with an exactly specified composition and fabrication (Ellson *et al.*, 2003 ▸). There is currently no purpose-built, acoustically transparent crystallization plate that is constructed from acoustically compatible (impedance-matched) plastic. We have previously demonstrated that protein crystals can be grown in and harvested from acoustically compatible 384-well polypropylene microplates (Labcyte Inc., Sunnyvale, California, USA) that can be adapted for protein crystallization (Cuttitta *et al.*, 2015 ▸). However, it would be more convenient to grow crystals in conventional crystallization labware such as the MiTeGen *In Situ*-1 crystallization plates used in this work. Even though the MiTeGen crystallization plates are not constructed from materials that are designed to be acoustically transparent, this approach is possible because their plastic bases are sufficiently thin that they do not obstruct acoustic ejection.

Improvements in crystal-handling automation have reduced cryogenic auto-mounter duty cycles to <3 min per crystal at most facilities, with some robotic auto-mounters approaching 1 min per crystal (Nurizzo *et al.*, 2016 ▸; Snell *et al.*, 2004 ▸; Soltis *et al.*, 2008 ▸; reviewed in Wasserman *et al.*, 2015 ▸). Harvesting crystals at comparable rates is challenging, particularly for high-throughput synchrotron experiments such as diffraction-based fragment screening (Viola, Carman, Walsh, Frankel *et al.*, 2007 ▸).^2^ A review of protein crystal-harvesting approaches categorized different technologies based on the level of human involvement and on the extent of contact between the harvesting apparatus and the specimen (Deller & Rupp, 2014 ▸). Robotic technologies require a harvesting tool that contacts the specimen, and these robots are becoming increasingly operator-independent (Viola, Carman, Walsh, Miller *et al.*, 2007 ▸). Commercially available crystal-harvesting devices claim a throughput of >1 harvest per minute. Contact-free crystal harvesting is possible using laser tweezers (Wagner *et al.*, 2013 ▸) or magnetic convection (Tung *et al.*, 2014 ▸) with the assistance of experienced human operators. Liquid-handling-assisted harvesting is a promising alternative for minimizing solvent background in cases where the crystals are sufficiently robust to endure the solvent-removal process (Luft *et al.*, 2014 ▸; Kitago *et al.*, 2010 ▸). In cases where room-temperature diffraction data are advantageous, microfluidic traps (Lyubimov *et al.*, 2015 ▸) or silicon chips (Owen *et al.*, 2017 ▸) can leverage one harvest step into many diffraction experiments. Recently, an automated magnetic manipulator-based crystal-harvesting system with a duty cycle of 2.4 min per specimen was described (Zeydan *et al.*, 2017 ▸). This harvesting time per crystal is comparable to photo-ablation harvesting (Zander *et al.*, 2016 ▸) and robotic harvesting (Viola, Carman, Walsh, Miller *et al.*, 2007 ▸).^2^

However, the greatest need for rapid crystal harvesting has come from serial crystallography and combinatorial crystallography (here, we use serial crystallography to mean the assembly of one data set from many crystals and combinatorial crystallography to mean the assembly of many data sets from chemically perturbed crystals). This is particularly true where one sample holder can contain many samples (so that the complete data-set rate may exceed the auto-mounter duty cycle; Roedig *et al.*, 2015 ▸). The availability of ∼5 µm mini-beam facilities and ∼1 µm micro-beam facilities (Smith *et al.*, 2012 ▸) with precision instrumentation (Hirata *et al.*, 2016 ▸) optimizes the use of multiple crystals per sample holder (Baxter *et al.*, 2016 ▸). In particular, drug-discovery applications such as high-throughput fragment screening could be greatly accelerated if the assay throughput were limited by the X-ray brilliance (<1 s per data set at third-generation synchrotrons) rather than by the crystal-harvesting speed or by the auto-mounter duty cycle. Hence, there is a growing need for crystal-harvesting techniques that are fast enough to keep up with high-brilliance X-ray facilities, can reduce the background to exploit micro-beams and mini-beams, and can combine harvested crystals with screened chemicals.

Here, we explore the viability of acoustically harvesting crystals from the crystallization plate in which they were grown, and we test several conventional plate designs to identify characteristics that would be desirable in a purpose-built acoustically harvestable crystallization plate. We demonstrate that a commercially available Echo 550 liquid-handling instrument (Labcyte Inc., Sunnyvale, California, USA) can be used to harvest protein crystals from slightly modified MiTeGen *In Situ*-1 plates. We further demonstrate that crystals grown in this plate can be serially harvested and then combined with chemicals from a fragment library. Using this technique, 96 crystal aliquots were harvested, immediately soaked with a fragment library and then cryocooled in under 1.5 h. This was reduced to less than half an hour when a six-axis robot was used to cryocool the acoustically harvested crystals. By comparison, manual specimen preparation required between 3 and 9 h for 96 specimens, depending on the skill of the experimenter and the required workflow (see Supplementary Table S1). Furthermore, ADE is ideal for handling chemicals at high concentrations (including above the solubility limit). ADE is particularly suited to harvesting small crystals that require reduced soaking times, and it prevents crystals from disintegrating owing to osmotic stress.

Initial testing was performed using unmodified crystallization plates, and the harvesting process was laborious and clumsy because crystallization plates are not designed with acoustic compatibility in mind. This necessitated the fabrication of a hybrid plate ‘assembly’ that contained some components from an Echo-compatible plate (to satisfy the plate-verification step performed by the Echo 550) and some components from conventional crystallization plates (to grow the protein crystals). For most applications, it is likely that the convenience of acoustic crystal harvesting does not justify the effort required to assemble a hybrid plate ‘assembly’. A more straightforward approach is required. To this end, we examined an assortment of commercially available crystallization plates in order to identify one that is appropriate for acoustic harvesting with no added components. We found that MiTeGen *In Situ*-1 crystallization plates are suitable for acoustic crystal harvesting with minimal modification that is readily achievable by lightly abrading the edge pedestal using sandpaper. The 15 min abrasion procedure is described in detail (see §[Sec sec2.1.3]2.1.3; acoustically compatible MiTeGen *In Situ*-1 crystallization plates are available from the correspondence authors on request). Modified plates can be used for both the manual and automated setup of crystallization assays. Once crystals have formed, the crystals can be either individually or serially harvested using a commercial Echo 550 liquid handler. In the discussion, we propose modest technical improvements that could make this crystal-harvesting system simpler to use. The most important improvement is to design an acoustically compatible crystallization plate. Crystal visualization inside the acoustic injection apparatus would also be helpful.

## 2. Materials and methods   

To demonstrate crystal harvesting from non-acoustic labware, we used the Echo 550 to transfer five types of test protein crystals (thermolysin, lysozyme, trypsin, proteinase K and ferritin) onto micro-meshes. The crystals were harvested from modified MiTeGen *In Situ*-1 plates. Thermolysin, lysozyme, trypsin and proteinase K were used for proof of principle because they can be grown in a variety of conditions and sizes that are simple to harvest acoustically. We chose ferritin as a test protein that presents harvesting challenges similar to those of expressed proteins (delicate crystals that are few in number in a mother liquor that contains a skin).

To demonstrate acoustic crystal harvesting from non-acoustic labware, crystals of thermolysin, lysozyme, trypsin, proteinase K and ferritin were grown using conventional hanging-drop techniques (Table 1 ▸) on MiTeGen plates that are suitable for *in situ* data collection^3^ (crystallization protocols are described in Supporting information §S1). The crystallization protocol for lysozyme crystals and for the proteinase K crystals that were used for fragment screening was modified such that the crystals were suspended in a Bingham fluid by adding 0.15% agarose (see Supplementary Fig. S2).

### 2.1. Fabricating and testing plates for acoustic crystal harvesting   

Two conditions are necessary to harvest protein crystals from a crystallization plate: (i) the plate bottom material must allow the propagation of sound and (ii) the transducer in the Echo 550 must be positioned at a precise focal distance from the crystals to be harvested. Our goal was to determine a plate that could achieve this with minimal modification (Supplementary Fig. S1). A flexible custom plate assembly (§[Sec sec2.1.1]2.1.1) was used to test the viability of acoustically harvesting crystals from many commercially available crystallization plates (five of which proved to be suitable for detailed acoustic investigation; §[Sec sec2.1.5]2.1.5). A second custom plate assembly (§[Sec sec2.1.2]2.1.2) was used to further test the most promising plate, and in particular to demonstrate that protein crystals could be harvested from the plate in which they were grown (rather than separately grown and then transferred). Finally, a simple 15 min procedure was developed that allows the most promising plate to be used for acoustic crystal harvesting with no additional components or assembly (§[Sec sec2.1.3]2.1.3).

For both of the plate assemblies (§§2.1.1 and 2.1.2), agarose was used to couple components from crystallization plates to components from acoustically compatible plates. To prepare the agarose pillow that is used to couple the labware, 1% agarose (Sigma–Aldrich, catalog No. A6877) was heated in deionized water to 100°C for 1 min. A 1000 µl pipette was used to fill each well in the polypropylene honeycomb structure with agarose. An additional 2.0 mm layer of agarose was carefully layered on top of the honeycomb, taking care to prevent bubbles. The non-acoustic labware was pressed into the agarose layer (while the agarose was still liquid) until it contacted the honeycomb structure (except for the experiment described in §2.2, where the agarose was set before the plate fragments were added).

#### 2.1.1. Fabricating a polypropylene assembly   

**Figure 2:**
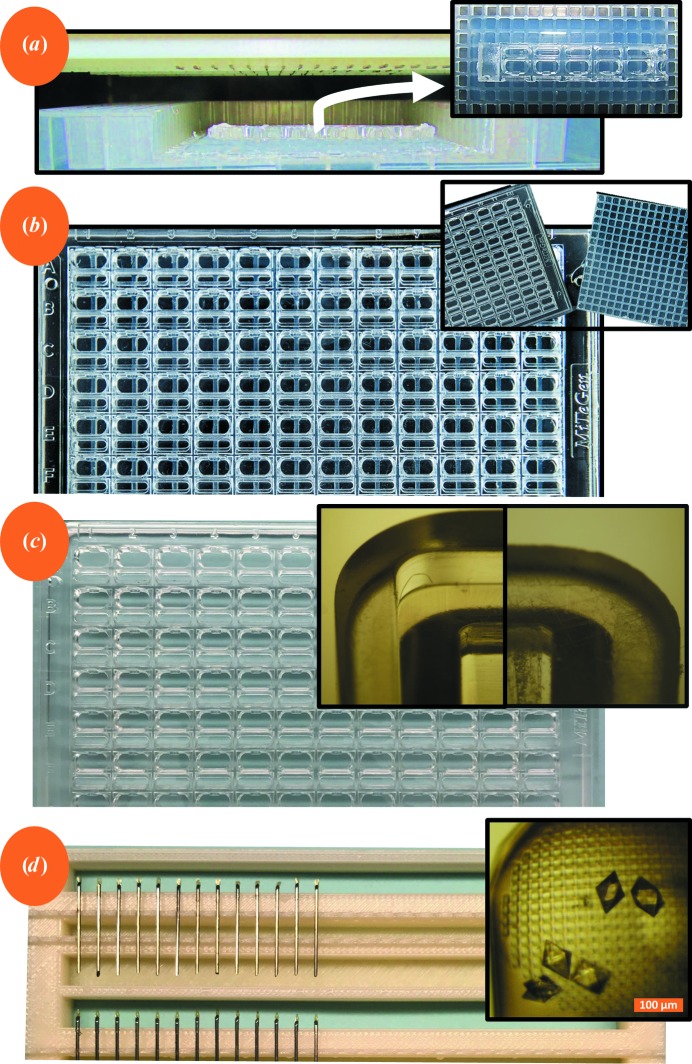
Overview of the apparatus for acoustic ejection from non-acoustic labware. For acoustic ejection of protein crystals to take place, the distance between the acoustic transducer in the Echo 550 and the bottom of the crystallization plate must equal one of two possible preset values. To accomplish this, crystallization plates must either be cut and placed on a plate of the correct height (*a*), be combined with a spacer (*b*) or be lightly sanded (*c*). There are no similar limits to the size of the destination plate (*d*). (*a*) For initial testing, the Echo 550 was used to harvest crystals from a polypropylene assembly which contained fragments of different commercially available plates (inset). (*b*) To test the harvesting of crystals grown inside the most promising crystallization phase, it was coupled to a thin slice from an acoustically transparent plate (inset). (*c*) Finally, intact MiTeGen plates were used to grow and harvest protein crystals by lightly sanding down the edge pedestal (inset). (*d*) Acoustically harvested crystals were transferred to a pin platform that contained up to 96 micro-meshes. The crystals on the micro-meshes (inset) were then pressure-fitted into a MiTeGen Reusable Base (model B1A-R) and cryocooled.

Five commonly used crystallization plates were cut into pieces that were one crystallization chamber wide and five crystallization chambers long (hereafter referred to as ‘plate fragments’). These plate fragments were used to determine whether the MiTeGen *In Situ*-1 plate is the most suitable for acoustic harvesting (Fig. 2 ▸ *a*, inset; a detailed description of plate modification is given in Supporting information §S2). This apparatus was used both to examine the acoustic properties of non-acoustic labware (see §[Sec sec2.1.5]2.1.5) and to harvest crystals from the plate fragments, either one crystal at a time (see §2.2) or rapidly for high-throughput screening (see §§2.3 and 2.4). Separately grown crystals (some containing colorants) were transferred to the plate fragments and then acoustically harvested onto micro-meshes (several crystals per mesh) for X-ray data collection (Fig. 2 ▸ *d*).

#### 2.1.2. Fabricating a MiTeGen assembly   

Once it had been determined that the MiTeGen *In Situ*-1 plate was a good candidate for acoustic harvesting, an intact plate (rather than plate fragments) was tested by coupling it to a spacer. The spacer was needed to optimize the distance between the bottom of the plate and the transducer in the Echo 550 (hereafter referred to as the ‘MiTeGen assembly’; a detailed description of the assembly is given in Supporting information §S2). The MiTeGen assembly was used to grow protein crystals and then to harvest those crystals onto micro-meshes for X-ray data collection (§[Sec sec2.1.5]2.1.5).

#### 2.1.3. Fabricating an acoustically compatible MiTeGen plate   

MiTeGen *In Situ*-1 crystallization plates were modified (Fig. 2 ▸ *c*) by abrading the 1.22 mm edge pedestal from each plate (Fig. 2 ▸ *c*, inset). The abrasion was performed using 100 grit sandpaper for approximately 10 min and then using wet 320 grit sand paper for 5 min to smooth the plate and completely remove the edge pedestal (see Supplementary Fig. S3). The abrasion process can cause the plate to become warped so that it does not sit evenly in the loading dock of the Echo 550. A warped plate can still be used for acoustic harvesting if moderate downward pressure is placed on the plate as it is loaded into the Echo 550.

#### 2.1.4. Acoustic properties of non-acoustic labware using the polypropylene assembly   

The Echo *WellPing* software (Labcyte Inc., Sunnyvale, California, USA) was used to examine the acoustic properties of five commonly used crystallization plates. Each design was placed on an agarose pillow that was deposited on a polypropylene plate with a honeycomb structure that was machined down to 1.7 mm, as described in §[Sec sec2.1.1]2.1.1 (Fig. 2 ▸ *a*). The acoustic signature of each of the plate designs was recorded. These data were used to select two plates that were suitable for acoustic harvesting (MiTeGen and CrystalDirect). Owing to their availability and physical strength, MiTeGen plates were used for all crystal-harvesting tests described here. However, crystals were also harvested from a polypropylene assembly containing CrystalDirect plate segments, and it is likely that CrystalDirect plates would be suitable for acoustic harvesting with minimal modifications similar to those described in Supplementary Fig. S3 (data not shown).

#### 2.1.5. X-ray diffraction from acoustically harvested crystals   

Diffraction data from crystals harvested using the polypropylene assembly (§[Sec sec2.1.1]2.1.1) were collected on beamline X25 at the National Synchrotron Light Source (NSLS). Diffraction data from crystals harvested using the MiTeGen assembly (§[Sec sec2.1.2]2.1.2) were collected on beamline A1 at the Cornell High Energy Synchrotron Source (CHESS) and beamline BL14-1 at the Stanford Synchrotron Radiation Lightsource (SSRL). Diffraction data from crystals harvested using the MiTeGen plate (§[Sec sec2.1.3]2.1.3) were collected on beamline 17-ID-1 (AMX) at the National Synchrotron Light Source II (NSLS II). Lysozyme and thermolysin data sets were processed with *HKL*-2000 (Otwinowski & Minor, 1997 ▸), and proteinase K data sets were processed with *XDS* (Kabsch, 2010 ▸). Data were further processed using *CTRUNCATE* in the *CCP*4 suite (Winn *et al.*, 2011 ▸). Structures were obtained by molecular substitution from published models and were refined using *REFMAC* (Winn *et al.*, 2003 ▸) and *ARP*/*wARP* (Lamzin & Wilson, 1993 ▸). The starting model PDB codes were 4tln for thermolysin (Holmes & Matthews, 1982 ▸), 1lyz for lysozyme (Diamond, 1974 ▸), 4ncy for trypsin (Yin *et al.*, 2014 ▸) and 4fon for proteinase K (Jakoncic *et al.*, 2006 ▸). The structures were visually inspected using *Coot* (Emsley & Cowtan, 2004 ▸).

### 2.2. Acoustically harvesting protein crystals from a polypropylene assembly   

All crystal-harvesting trials using the polypropylene assembly were carried out with crystals that were separately grown by the conventional hanging-drop method and then transferred into the MiTeGen plate fragment prior to harvesting trials.

**Figure 3:**
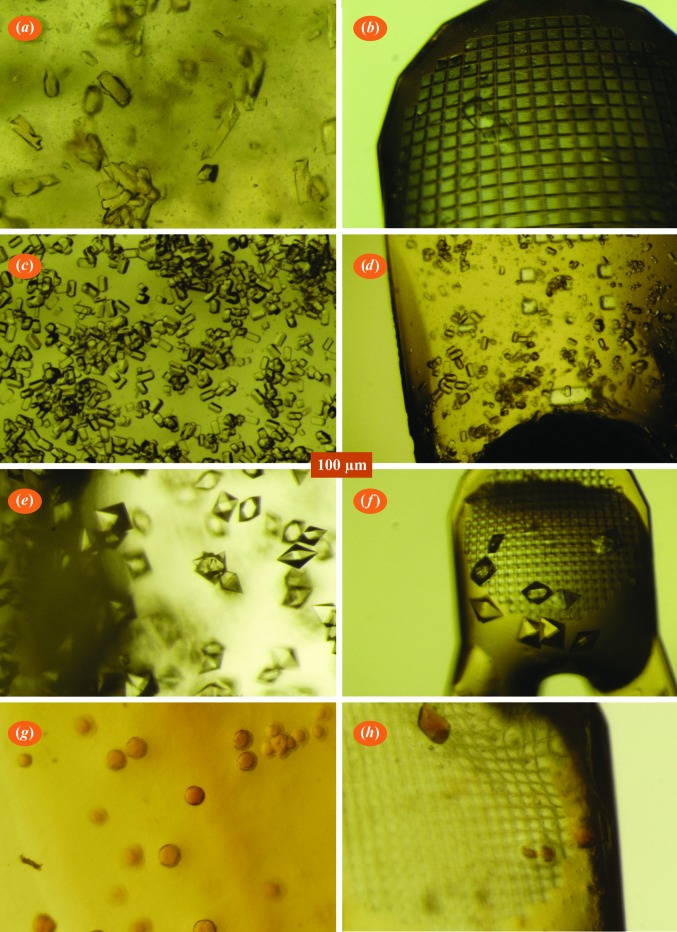
Acoustic crystal harvesting. (*a*, *b*) Thermolysin crystals in a polypropylene assembly (*a*) harvested onto a micro-mesh (*b*). (*c*, *d*) Lysozyme crystals grown in a MiTeGen assembly (*c*) harvested onto a micro-mesh (*d*). (*e*, *f*) Proteinase K crystals grown in a MiTeGen plate (*e*) harvested onto a micro-mesh (*f*). (*g*, *h*) Ferritin crystals grown in a MiTeGen plate (*g*) harvested onto a micro-mesh (*h*). Only a few thermolysin crystals were present in each harvested aliquot, and there were occasional cases where no crystals were observed on the micro-mesh. Lysozyme and proteinase K crystals were harvested in much greater numbers and no harvesting failures were observed.

In order to compare controls with crystals acoustically harvested from a polypropylene assembly, the apparatus described in §[Sec sec2.1.1]2.1.1 was assembled with MiTeGen plate segments containing thermolysin crystals. Ten thermolysin crystals were acoustically transferred to micro-meshes. Additionally, ten thermolysin crystals were hand-transferred onto cryoloops (a typical harvested crystal is shown in Fig. 3 ▸ *b*). All crystals were cryocooled. X-ray diffraction data were obtained from each of the acoustically harvested test crystals and similarly from each of the hand-harvested control crystals.

The polypropylene assembly (§[Sec sec2.1.1]2.1.1) was used to test the viability of harvesting a specific protein crystal (this would be useful if an acoustic harvesting system could be fitted with an internal microscope). A Leica microscope with a polarizing lens was used to discover the locations of promising trypsin crystals in a polypropylene assembly (Fig. 2 ▸ *a*) containing a MiTeGen plate fragment (the plate fragment could slide over the cool agarose pillow). The trypsin crystals were colored with a red dye for clarity. After a crystal had been selected for harvesting, its position was adjusted by sliding the MiTeGen plate section over the agarose pillow until the center of the crystal was aligned with the center of one of the wells in the polypropylene assembly. The level of wetness of the agarose pillow was balanced so that there was a good acoustic coupling to the crystal selected for harvesting, while it was not so wet that the non-acoustic labware would inadvertently slide out of position. A pin platform was fitted with micro-meshes (Fig. 2 ▸ *d*).

The polypropylene assembly was placed in the source tray of the Echo 550. The pin platform was placed in the destination tray. The Echo *ArrayMaker* software (Labcyte Inc., Sunnyvale, California, USA) was then used to harvest the desired crystal out of the MiTeGen plate fragment and onto the designated micro-meshes in the pin platform. The micro-mesh containing the crystal was then manually removed from the pin platform, inserted into a MiTeGen Reusable Base (model B1A-R) and immediately cryocooled in liquid nitrogen (inserting a pin into a reusable base and cryocooling it takes <5 s).

For high-throughput screening of fragment libraries using a polypropylene assembly, the apparatus described in §[Sec sec2.1.1]2.1.1 was assembled with MiTeGen plate segments containing lysozyme crystals. The lysozyme crystals had a cuboidal habit with a long axis of approximately 50 µm. The plate segment contained 25 µl of dense crystal slurry with a concentration of approximately 100 crystals per microlitre. A pin platform was assembled as described above, but in this case the pin platform was populated to its full capacity with 96 micro-meshes. A polypropylene source plate was prepared containing a mini-library of 33 chemicals, including two known lysozyme ligands: *N*-acetylglucosamine (NAG) and benzamidine. The Echo 550 was used to dispense 10 nl of each of the chemicals in the library into a distinct micro-mesh. The solvent around each chemical was allowed to evaporate (leaving the chemical residue adhered to the micro-mesh). The Echo 550 was then used to transfer 25 nl of lysozyme crystal slurry to 36 micro-meshes (including three controls without chemicals). All of the crystal-containing micro-meshes were cryocooled and X-ray diffraction data were individually obtained from each specimen.

### 2.3. Acoustically harvesting protein crystals grown on a MiTeGen assembly   

The apparatus described in §[Sec sec2.1.2]2.1.2 was used to harvest lysozyme crystals that were grown directly on a MiTeGen assembly (rather than separately grown and then transferred, as described in §[Sec sec2.2]2.2). Eight lysozyme crystals were acoustically harvested onto micro-meshes (similar control crystals were manually harvested onto cryoloops). A typical harvested crystal is shown in Fig. 3 ▸(*d*). X-ray data were obtained from both the acoustically harvested and control lysozyme crystals.

### 2.4. Acoustically harvesting protein crystals grown on a MiTeGen plate   

The apparatus described in §[Sec sec2.1.3]2.1.3 was used to harvest crystals that were grown directly on the MiTeGen plate (rather than separately grown and then transferred, as in §[Sec sec2.2]2.2). Crystals of proteinase K, lysozyme and ferritin were grown and then harvested (Figs. 3 ▸ *e*–3 ▸ *h*). The Echo 550 settings required to harvest crystals from modified MiTeGen *In Situ*-1 crystallization plates are detailed in Supplementary Table S2 (and are illustrated in Supplementary Fig. S5). It was observed that the average number of crystals harvested from a given drop decreases with each ejection from a well containing crystals in mother liquor. In contrast, the average number of crystals harvested from a well containing crystals suspended in a Bingham fluid remains constant. In high-throughput screening applications, it is advantageous to perform many serial ejections with an equal number of crystals harvested each time. Since proteinase K and lysozyme were used for high-throughput chemical library screening, all of the crystals described in this section were grown in a Bingham fluid (as described in Supplementary Fig. S2). Ferritin crystals were not used for high-throughput screening and were not in a Bingham fluid.

To use sound to set up a MiTeGen plate and to harvest crystals from that plate, DropSaver lids (Zipper *et al.*, 2014 ▸) were fastened onto a modified MiTeGen plate, and the Echo 550 was used to dispense proteinase K and Bingham precipitant (as described in Supplementary Fig. S2). Crystals were grown in 12 wells of the plate, with the total drop volume ranging from 1000 to 3200 nl in 200 nl increments. The crystals were left to grow overnight. To determine the minimum drop volume needed for acoustic harvesting, ejection of protein crystals was attempted from each drop.

To compare controls with crystals acoustically harvested from a MiTeGen assembly, the apparatus described in §[Sec sec2.1.3]2.1.3 was used to harvest proteinase K crystals that were grown directly on a MiTeGen plate (rather than separately grown and then transferred into the plate, as described in §[Sec sec2.2]2.2). Ten proteinase K crystals were acoustically harvested onto micro-meshes (similar control crystals were manually harvested). X-ray data were obtained from both varieties of proteinase K crystals.

After a crystal has been acoustically harvested, it can be rapidly combined with a chemical from (for example) a fragment library. The time and effort needed to harvest crystals for use in chemical library screening projects has driven efforts to use acoustic methods to improve the workflow for crystal growth (Wu *et al.*, 2016 ▸), crystal harvesting (Chen *et al.*, 2004 ▸) and chemical dispensation (Collins *et al.*, 2017 ▸). Modified MiTeGen plates were used to explore simultaneous acceleration of crystal growth, crystal harvesting and chemical dispensation. Lysozyme crystals were grown in a Bingham fluid (as described in Supplementary Fig. S2) in one well of a MiTeGen plate. Acoustic pulses were used to serially harvest the lysozyme crystals onto a pin platform containing 96 micro-meshes. Colored dyes were then added to the lysozyme crystals. The first six micro-meshes containing crystals and dye were photographed to demonstrate that each harvested crystal was correctly paired with its intended dye.

Sound waves can impart momentum to either liquids or suspended solids. Consequently, acoustic methods are suitable for high-throughput screening applications involving high-concentration chemical libraries. To demonstrate this, proteinase K crystals were screened against a mini-fragment library of chemicals at 200 m*M* concentration (including supersaturated solutions and suspended solids in cases where the solubility was less than 200 m*M*).^4^ The technique described above was used to serially harvest proteinase K crystals onto 96 micro-meshes and then to combine these crystals with 96 non­hazardous chemicals in a small fragment library. The crystals were soaked with the chemicals for 10 min. X-ray diffraction data were obtained from each of the 96 soaked crystals.

Throughout this research, it was observed that when multiple crystal aliquots are harvested from a single crystallization well, each successive aliquot contains fewer crystals. Although this problem is likely to be innocuous for single structure projects, it is highly problematic in cases where many aliquots must be harvested during the course of one experiment. This includes combinatorial crystallography projects (such as high-throughput fragment screening), which are most likely to benefit from acoustic crystal harvesting. Many attempts were made to overcome this problem before adding a low concentration of agarose (usually about 0.2%) to both the protein buffer and the crystallization cocktail was tried. This induces the crystals to grow in a Bingham fluid, which acts like an ejectable fluid during crystal setup and harvesting, but otherwise acts like a gel that prevents the movement of crystals within the fluid. To test the effectiveness of this strategy, we serially harvested 25 nl of proteinase K in a Bingham suspension and visually counted the number of crystals in each ejected aliquot as a function of the number of harvests.

To describe the trajectory of crystals moving towards the ejection point, ten stacks of bright-field images were obtained, each consisting of ten images with evenly spaced focal points. Between each stack a single 10 nl aliquot of proteinase K crystals was harvested as described in Supplementary Fig. S2. For each of the bright-field stacks, custom software was then used to generate a three-dimensional model to help to visualize the locations of all crystals beneath the ejection point (Gofron *et al.*, 2018 ▸). Custom object-tracking software was employed to generate a model for the trajectory described by each proteinase K crystal as it approached the crystal-harvesting point.

## 3. Results   

Assembly and testing of the polypropylene assembly (§[Sec sec2.1.1]2.1.1) and the MiTeGen assembly (§[Sec sec2.1.2]2.1.2) were time-consuming. The fit between each component had to be individually adjusted before acoustic crystal harvesting was possible (for example the fit between the plate segments and the polypropylene assembly). In contrast, harvesting crystals from the modified MiTeGen plate (§[Sec sec2.1.3]2.1.3) was straightforward and reliable.

### 3.1. Acoustic signature of non-acoustic labware   

**Figure 4:**
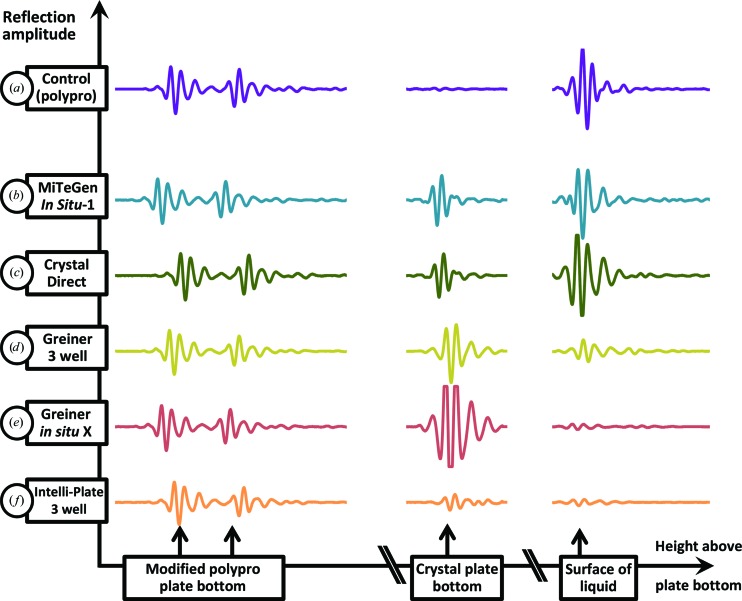
Acoustic signatures of diverse plates. The Echo 550 was used to ‘ping’ each of the polypropylene (polypro) assemblies and to record the acoustic echo from the components of each assembly. The acoustically compatible polypropylene source plate (*a*) exhibits two modest reflections from the plate bottom (left) and a strong reflection at the liquid–air interface (right). This strong pulse is needed for crystal ejection. The MiTeGen *In Situ*-1 plate (*b*) and the CrystalDirect plate (*c*) reflected a modest amount of energy (middle) but sufficient power was retained at the surface to eject crystals. In contrast, three plate designs experienced an excessive loss of energy and there was insufficient acoustic power at the surface to eject crystals. The two Greiner plates (*d*, *e*) lost significant energy through reflection. In contrast, scattering must account for most of the power loss in the Intelli-Plate (*f*) since there were no audible reflections.

The sound pulse must retain sufficient amplitude in order to eject crystals from the crystallization drop. The amplitude is reduced mainly by scattering (loss of energy inside a bulk material) and by reflection (loss of energy at the interface between two materials). Both of these sources of energy loss played a role in making one or more of the tested plates unable to eject crystals. Fig. 4 ▸ shows representations of the energy reflected from all of the interfaces in each tested non-acoustic labware, as well as a control measurement with no plate segment present. The acoustic energy reflected from the non-acoustic labware is directly detected by the Echo 550. The scattered energy can be computed by comparing the amplitude of the reflection from the liquid–air interface with the amplitude obtained when no plate is present. All reflections were scaled using the intensity of the reflection from the bottom of the modified polypropylene plate (since this component is common to all of the tested systems and occurs before any of the other reflections). The results confirm that the MiTeGen plate and the CrystalDirect plate are acoustically transparent. A custom-built acoustically transparent crystallization plate would be likely to perform even better.

### 3.2. Crystals can be acoustically harvested from a polypropylene assembly   

The majority of the testing for this project was carried out using the hybrid polypropylene assembly described in §[Sec sec2.1.1]2.1.1. Once a polypropylene plate had been modified to accept fragments from crystallization plates, it was possible to test many different plate designs and many variations of the acoustic harvesting strategies using this assembly. Since these tests were only relevant to the overall conclusion (that minimally modified MiTeGen *In Situ*-1 plates are suitable for acoustic harvesting), most of the details are not described here (a full description is given in Supporting Information §S3).

Plate fragments were coupled to the polypropylene plate as described in §[Sec sec2.1.1]2.1.1. In some cases, acoustic harvesting was not possible because too much acoustic energy was lost (see §[Sec sec3.1]3.1) so that the momentum transferred to the crystal slurry was insufficient to eject a droplet. However, MiTeGen plates and CrystalDirect plates did not greatly diminish the acoustic signal, and crystals were harvested from both. Control crystals were manually harvested. Diffraction data from acoustically harvested crystals were similar to diffraction data from manually harvested controls (Table 1 ▸; see Supporting information §S3 for full details).

**Figure 5:**
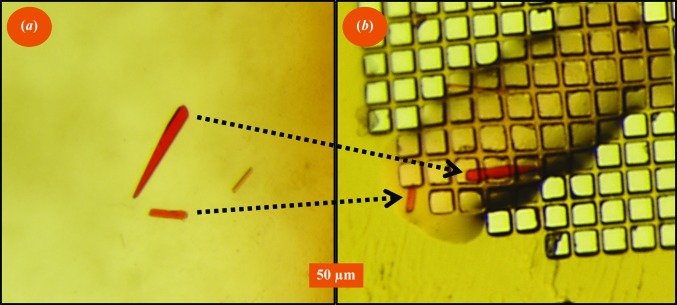
Click to mount: ejecting a selected crystal cluster. We selected a cluster of three crystals and carefully aligned these crystals with the ejection zone. We then used the Echo 550 to harvest these crystals onto a micro-mesh. (*a*) shows a view of the crystallization well; (*b*) shows a micro-mesh image.

To determine whether it was possible to acoustically harvest one specific crystal, a small crystal cluster containing two moderate-sized trypsin crystals was targeted, and these specific crystals were ejected onto a micro-mesh (Fig. 5 ▸). The successful ejection of specifically targeted crystals required careful alignment of the crystals with the ejection zone (there were many near-misses). This process would be greatly simplified if the Echo 550 had an internal visualization system.

The mini-library that was combined with lysozyme contained 33 common laboratory chemicals that had no significant hazards. The average molecular weight of our mini-library chemicals was 159 g mol^−1^ and the average molecular volume was 134 Å^3^; the average *c*log*P* was −2.08. The nominal concentration of the chemicals was 200 m*M* (chemicals with low water solubility were ejected as supersaturated solutions or suspended solids). The only chemicals that were observed to bind to lysozyme were NAG and benzamidine (Supplementary Fig. S4).

### 3.3. Crystals grown in a MiTeGen assembly can be acoustically harvested   

The X-ray data obtained from eight lysozyme crystals that were acoustically harvested (from the MiTeGen assembly in which they were grown) were at a slightly lower resolution (Δ_resolution_ = 0.08 Å) compared with the data from similar hand-harvested crystals (Table 1 ▸; see Supporting information §S4 for full details and Supplementary Table S4 for the full data).

### 3.4. Crystals grown in a modified MiTeGen plate can be acoustically harvested   

The simple modifications that are needed to enable acoustic harvesting from a MiTeGen plate might be worthwhile for projects that require a single diffraction data set from one or a few isomorphous crystals. However, combinatorial crystallo­graphy is an obvious application since these modifications enable multiple acoustic harvests from the same plate, and further enable each harvested aliquot to be combined with distinct chemicals. To determine the minimum drop volume needed for acoustic harvesting, ejection of protein crystals within varied drop volumes was attempted.

To identify the minimum crystallization drop volume for acoustic harvesting, 12 drops of increasing volume were set up (1000 nl + *N* × 200 nl) and it was observed that acoustic harvesting was reliable from drops with a minimum volume of 1800 nl. This 1800 nl ‘dead volume’ has implications for setting up crystallization drops. If an investigator wishes to harvest most of the prepared crystals, then a crystallization volume that is significantly greater than 1800 nl must be used.

The X-ray diffraction from four acoustically harvested proteinase K crystals was compared with the X-ray diffraction from four hand-harvested control crystals. As was the case with crystals harvested from a polypropylene assembly (§[Sec sec3.2]3.2), we observed no significant difference between the diffraction from acoustically mounted crystals and hand-mounted controls (Table 1 ▸). The mean resolution limit [*I*/σ(*I*) = 1.0] was 1.72 Å (*R*_merge_ = 11.0%) for acoustically harvested proteinase K crystals, compared with 1.80 Å (*R*_merge_ = 11.9%) for hand-harvested controls (Table 1 ▸).

**Figure 6:**
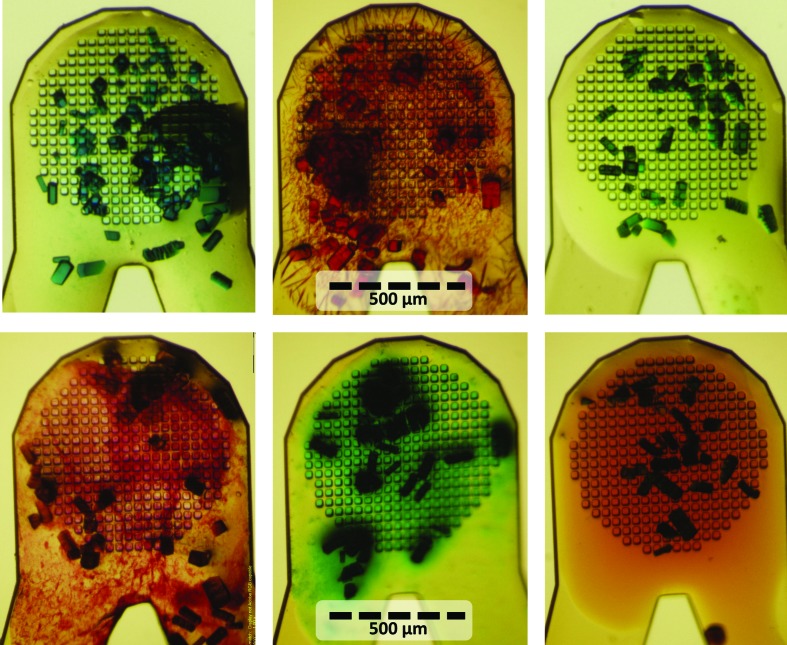
Harvested crystals are combined with colorants. Lysozyme crystals were harvested to six micro-meshes (25 nl aliquots) and combined with six different colorants (10 nl aliquots). Each colorant was observed correctly paired with its intended crystals (note that many of the colorants selectively penetrate into the lysozyme crystals).

One of the goals of this project was to demonstrate the synergy between acoustic harvesting and combinatorial crystallography. Lysozyme and proteinase K crystals were acoustically harvested and then acoustically combined with colorants. The colorants were observable using a simple bright-field microscope, demonstrating that each colorant was correctly paired with its intended crystal target (Fig. 6 ▸).

**Figure 7:**
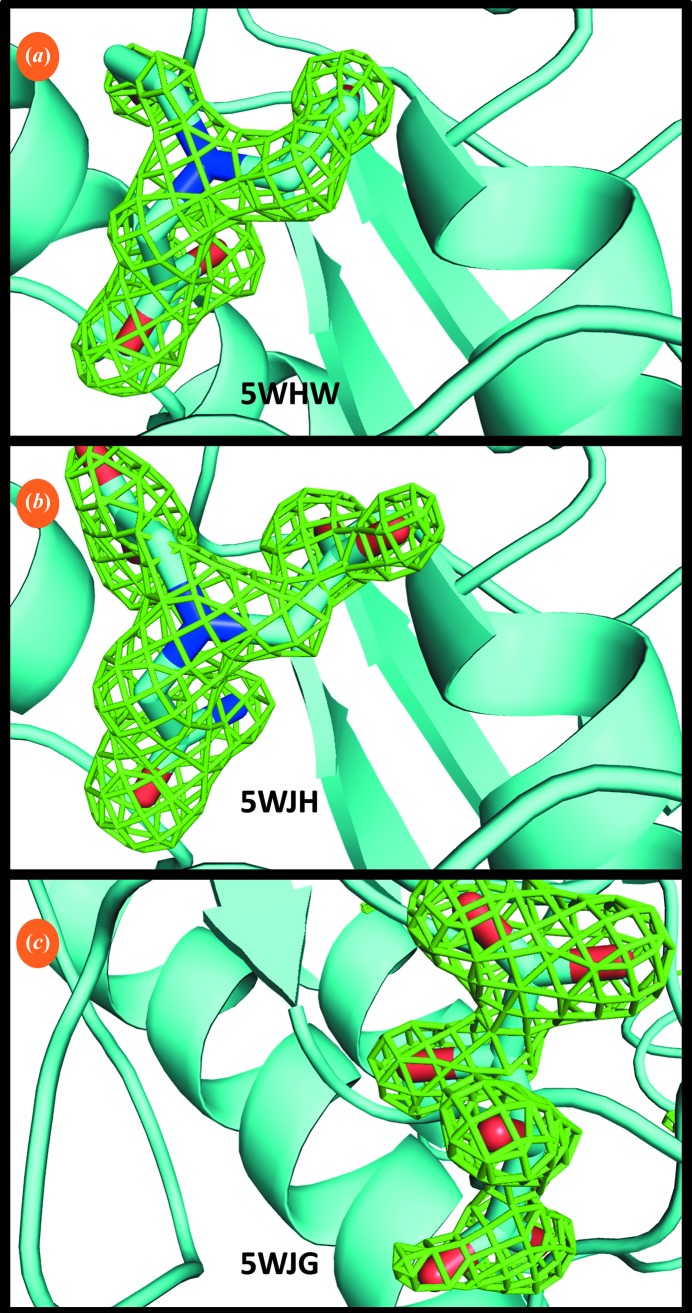
High-throughput fragment screening. Proteinase K crystals were rapidly screened against a fragment library consisting of 96 chemicals. The total laboratory preparation time was 4 min to set up the crystallization drop, 4 min to eject 96 crystal aliquots, 4 min to combine the drops with 96 screened chemicals and 72 min to cryocool the crystals and place them in pucks (this does not include overnight crystal growth or the 10 min soaking time). Three fragments bound to proteinase K were observed in the electron density (OMIT difference map contoured at 3σ; ligands were omitted from structure refinement). The structure-refinement statistics for bicine (*a*), *N*-(2-acetamido)iminodiacetic acid (ADA) (*b*) and tartrate (*c*) are shown in Table 2 ▸.

High-throughput applications (Magee, 2015 ▸) can benefit from acoustic workflow improvements that greatly increase the speed of soaking experiments. Proteinase K crystals were harvested onto 96 micro-meshes and immediately soaked with a nonhazardous 96-fragment screen. The entire procedure from placing the source plates into the Echo 550 to having 96 cryocooled screens ready in pucks required 1.5 h of time from two scientists. X-ray diffraction data were then obtained from many of the soaked crystals, and three previously unreported low-affinity ligands of proteinase K (Fig. 7 ▸, Table 2 ▸) were identified. Two of these would have been difficult to identify using conventional screening methods. Using conventional manual soaking methods,^5^ only one of the three fragments (bicine) could be identified. One attempt failed because the 400 µm proteinase K crystals disintegrated immediately when they contacted the 200 m*M* *N*-(2-acetamido)iminodiacetic acid (ADA) solution; the other attempt failed because 1 h of soaking time yielded a low occupancy for tartrate (we note that other groups have previously demonstrated that crystal stability and soaking efficiency are increased when chemicals are acoustically introduced compared with conventional hand-soaking; see Collins *et al.*, 2017 ▸). Also, co-crystallization only produced diffraction-quality crystals for one of the ligands. Because of these difficulties, the harvesting and soaking experiments using acoustic techniques were repeated with each of the three ligands, and the resulting X-ray diffraction confirmed the three expected binding fragments.

**Figure 8:**
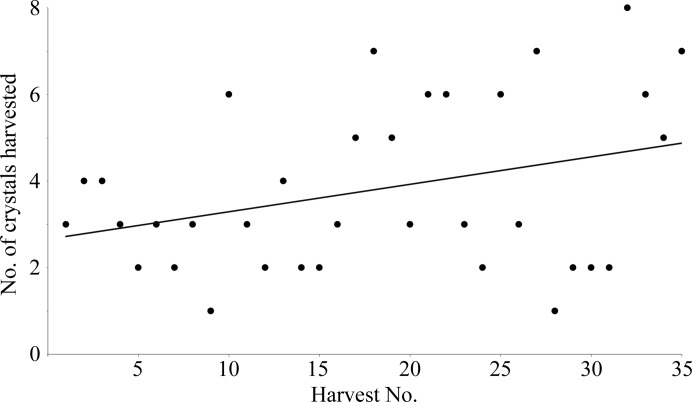
The number of crystals harvested from a Bingham fluid remains constant as additional aliquots are successively harvested from a single crystallization drop. The number of crystals in each 25 nl harvested aliquot is shown as a function of the number of successive harvests from a single crystallization well (the overall average was 3.8 ± 0.6 crystals per 25 nl harvest). The crystals in the source plate were grown in a Bingham fluid consisting of 0.15%(*w*/*w*) agarose (in addition to the normal crystallization components). The Bingham fluid crystallization protocol is described in Supplementary Fig. S2.

A key improvement that greatly simplified the workflow for acoustic combinatorial crystallography was to grow the protein crystals in a Bingham fluid. The Bingham fluid condition (addition of ∼0.2% agarose) keeps the number of crystals ejected more constant over successive pulses. The reason that this was so important is illustrated in Fig. 8 ▸, which demonstrates that acoustic harvests from a Bingham fluid yield a constant number of crystals per harvested aliquot.

**Figure 9:**
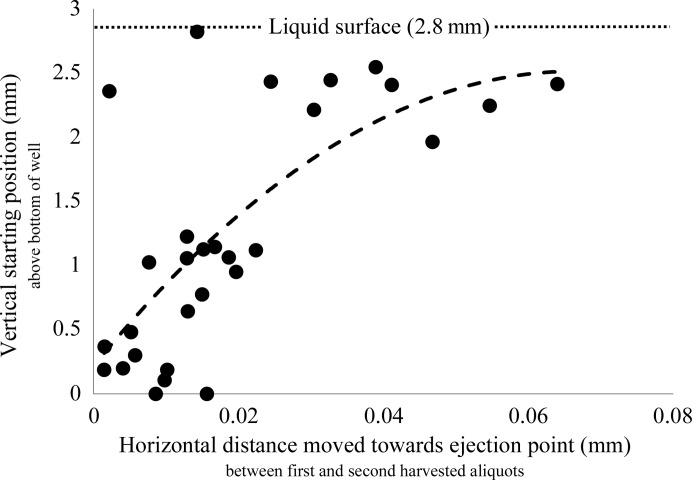
Harvested crystals are drawn from the surface layer. Observed movement towards the ejection point for all crystals in the field of view (caused by harvesting a 10 nl aliquot) as a function of the initial crystal depth. The movement of each crystal was determined by comparing two successive generated three-dimensional images of the crystal column suspended below the harvesting point (with the 10 nl harvest between them). Deep-dwelling crystals remained largely stationary during serial crystal harvesting, with the majority of the harvested crystals originating from surface layers that moved quickly towards the harvesting point. The data are fitted by a second-order polynomial (*y* = 520*x* ^2^ + 70*x*; one outlier was removed).

The sound pulses that are used to harvest protein crystals traverse the crystallization well in a narrow cone starting at the bottom of the well, and the expectation was that crystal trajectories would mirror this conical path (such that crystals located at the bottom of the solution would be pushed upwards). To test this, three-dimensional images of the crystal column beneath the ejection point were generated, crystals were then ejected and new three-dimensional images were generated (Fig. 9 ▸). This process was repeated ten times and custom software was used to generate a three-dimensional visualization of the trajectory taken by crystals moving towards the ejection point (Supplementary Fig. S2). By tracking the location of each crystal during successive ejections, it was shown that crystals residing near the surface moved rapidly towards the crystal-harvesting point, while crystals residing deep in the well remained largely stationary (Fig. 9 ▸). This finding demonstrates why serial acoustic crystal harvesting has proven to be difficult without the use of some artifact to prevent crystals from sinking into deep layers where they are not accessible to crystal harvesting.

## 4. Discussion   

Full automation of the high-throughput macromolecular crystal structure-determination pipeline would increase productivity in conventional structural biology, as well as enable novel discovery-based solutions to stubborn problems. Advances in automated protein production (Banci *et al.*, 2006 ▸), automated crystallization (Bolanos-Garcia & Chayen, 2009 ▸) and end-station automation (Snell *et al.*, 2004 ▸) have potentiated the goal of full automation, but crystal harvesting remains a stubborn bottleneck that prevents the output of crystallization facilities from matching the data-collection speeds available at next-generation synchrotrons (Berman *et al.*, 2011 ▸). In cases where very high speed is not required, robotic solutions (Viola, Carman, Walsh, Miller *et al.*, 2007 ▸), laser tweezer-assisted mounting (Wagner *et al.*, 2013 ▸), laser-assisted recovery on thin films (Cipriani *et al.*, 2012 ▸) and *in situ* methods on plates (Aller *et al.*, 2015 ▸) or microfluidic devices (Stojanoff *et al.*, 2011 ▸) are promising alternatives to manually harvesting individual crystals. In high-throughput applications such as fragment-library screening (Englert *et al.*, 2010 ▸) and automated proteomics (Manjasetty *et al.*, 2012 ▸) the speed of crystal harvesting must keep up with the fast serial data-collection methods that are being developed at synchrotrons (Chavas *et al.*, 2015 ▸) and X-ray free-electron lasers (Feld *et al.*, 2015 ▸).

Although the major focus of the work reported in this manuscript is to improve the harvesting and soaking workflow in combinatorial crystallography projects, the same technique could also be applied to crystal harvesting for serial crystallography applications. This is particularly true where one sample holder can contain either a few deposited samples (Yin *et al.*, 2014 ▸) or many patterned samples (Guo *et al.*, 2015 ▸). Structural proteomics has been accelerated by improvements in the upstream workflow, but often yields very small crystals (Manjasetty *et al.*, 2012 ▸). The limiting factor in microcrystallography is that the total usable diffraction from each crystal is limited by the dose limit that can be tolerated by the crystal (Owen *et al.*, 2006 ▸). Although laboratory techniques exist to slightly improve the dose tolerance (see, for example, Crosas *et al.*, 2017 ▸; Allan *et al.*, 2013 ▸), serial crystallography from microcrystals is usually the method of choice for overcoming the dose limit of small crystals.

We have previously demonstrated that acoustic methods can rapidly harvest crystals from plates that are optimized for acoustic transfer (Cuttitta *et al.*, 2015 ▸). In cases where crystals are already present on plates that are not optimized for acoustic transfer, fast serial harvesting may be attempted using hybrid plates similar to those described here. However, the hybrid plates were laborious to assemble and awkward to use. The slightly modified MiTeGen plate was intended to demonstrate a practical solution that allows a commercially available crystallization plate to serve as an acoustic harvesting platform. This also serves as a proof of concept in support of the eventual goal of an integrated acoustic harvesting system with purpose-designed crystallization labware. The most important improvement required is a crystallization plate that is designed for acoustic compatibility. High-throughput screening applications are a natural first fit for acoustic harvesting; small crystals are particularly suitable because they are easy to eject and because they combine rapidly with chemical libraries (Cole *et al.*, 2014 ▸). Our experience is that cuboidal crystals larger than 50 µm occasionally fail to eject, and crystals larger than 160 µm rarely eject (although much larger rod-shaped crystals can be ejected). Click-to-mount applications will benefit from improvements to the ADE hardware, such as an internal visualization system.

The ability to select single crystals for harvesting from conventional crystallization plates also has implications for the direct injection of crystals into the X-ray beam. We have previously demonstrated that crystals can be transferred from acoustically compatible plates onto a movable Kapton conveyor belt, which then transports the crystals into the X-ray beam (Roessler *et al.*, 2013 ▸). However, users may prefer to deliver specimens to the conveyor belt from familiar labware such as MiTeGen *In Situ*-1 plates. An X-ray end station equipped with an acoustic injection system could allow users to use beamline-control equipment to harvest their crystals from an acoustically transparent crystallization plate directly into the X-ray beam. A robotic plate-handling system such as the G-Rob (le Maire *et al.*, 2011 ▸) would suspend the crystallization plate face-down so that the back of the plate is in contact with the transducer that generates the crystal-harvesting sound pulse. Users would visually identify a desirable crystal and use a click-to-mount approach to eject the crystal onto a movable Kapton conveyor belt (alternatively, the acoustic system can directly detect each crystal using a sonar ‘ping’; Ericson *et al.*, 2016 ▸). The conveyor belt would translate the crystal into position for X-ray data collection, where it would be cryocooled in place using a gated cryostream.

Acoustic specimen preparation is particularly advantageous for operations at low volumes. Table 1 ▸ shows that small crystals (25 µm, lysozyme) yielded similar diffraction regardless of the harvesting technique, while large crystals (60 µm, thermolysin) yielded better data when hand-harvested (Δ_resolution_ = 0.3 Å), suggesting that acoustically harvesting larger samples imposes a trade-off between convenience and quality. For smaller samples, conventional handling is difficult and error-prone (Kong *et al.*, 2012 ▸). Acoustic transfer eliminates error owing to different liquids interacting in different ways with tips and tubing. Variations caused by the training and skill of individual human operators are also eliminated. Computer-operated harvesting of crystals limits the damage to crystals from physical contact with transfer materials (Tung *et al.*, 2014 ▸) and eliminates contaminants that may leach out of pipette tips, nozzles and plastic labware (McDonald *et al.*, 2008 ▸). Once a crystal has been transferred to its desired destination, additional components such as fragment libraries, heavy-metal solutions and cryoprotectants may be added to the same location. In such cases, touchless transfer prevents loss of the additive owing to adhesion to the surface of the transfer material (Harris *et al.*, 2010 ▸).

Acoustic crystal handling has the potential to accelerate the rate of specimen preparation to match the rate of specimen consumption at modern synchrotron X-ray sources. A fully automated structure-determination pipeline (including crystal harvesting and chemical handling) also allows researchers to carry out a high-throughput structure-based screen of protein crystals perturbed by a chemical library that probes the response to perturbations such as pH changes, water activity changes and (most of all) interactions with fragment libraries. This approach leverages the brightness of next-generation synchrotrons to generate families of related structures that explore how protein structure responds to environmental changes. Full automation will also ensure that the metadata for a project are generated by each instrument and then accurately transferred to the next instrument. Furthermore, automated crystal handling enables researchers to access a comprehensive shared chemical library archive (including fragment libraries, heavy atoms and cryoconditions).

## 5. Related literature   

The following reference is cited in the Supporting information for this article: Newman *et al.* (2009 ▸).

## Supplementary Material

PDB reference: proteinase K, 5whw

PDB reference: 5wjg

PDB reference: 5wjh

Supplementary Materials and Methods and Results and Supplementary Figures and Tables.. DOI: 10.1107/S2059798318011506/gm5056sup1.pdf

## Figures and Tables

**Table 1:** Crystallization conditions for acoustically harvested crystals The protein was dissolved in the indicated buffer, combined with an equal volume of the indicated precipitant and placed in vapor diffusion over the indicated reservoir. Note that the crystallization procedure used for high-throughput screening applications was adjusted such that the crystals were grown in a Bingham fluid, as described in Supplementary Fig. S2 (a detailed description is given in Supporting information §S1). Note that mean values for merging and refinement statistics are shown here (the statistics from individual refinements of thermolysin crystals are shown in Supplementary Table S3, those for lysozyme in Supplementary Table S4 and those for proteinase K in Supplementary Table S5).

Protein	Proteinase K		Lysozyme		Thermolysin	
Harvesting experiment						
Labware	MiTeGen *In Situ*-1 plate		MiTeGen *In Situ*-1 assembly		Polypropylene assembly	
Holder	Mesh	Mesh	Mesh	Loop	Mesh	Loop
Harvesting	Acoustic	Manual	Acoustic	Manual	Acoustic	Manual
Crystal size (µm)	50 × 50 × 50		25 × 10 × 10		60 × 20 × 20	
Crystallization conditions						
Protein (mg ml^−1^)	50		120		330	
Buffer	0.1 *M* bis-Tris pH 6.5		0.1 *M* sodium acetate pH 4.6		0.05 *M* Tris pH 7.5 + 45% DMSO	
Precipitant	0.8 *M* sodium nitrate + 0.08 *M* CaCl_2_		4% NaCl + 5% glycerol		1.45 *M* CaCl_2_	
Reservoir	1.6 *M* sodium nitrate + 0.16 *M* CaCl_2_		8% NaCl + 10% glycerol		Water	
Data-collection statistics						
No. of crystals	4	4	8	8	10	10
X-ray source	AMX, NSLS II		CHESS/SSRL		X25, NSLS	
Beam width × height (µm)	5 × 5		100 × 100		100 × 100	
Resolution (Å)	1.72 ± 0.27	1.80 ± 0.17	1.51 ± 0.11	1.43 ± 0.07	2.06 ± 0.32	1.73 ± 0.07
*R*_merge_ (%)	11.0 ± 2.1	11.9 ± 3.0	11.4 ± 3.4	6.5 ± 0.8	13.5 ± 3.9	7.7 ± 1.7
No. of reflections	24434 ± 8148	20581 ± 5382	19135 ± 3827	21032 ± 1900	33781 ± 7553	41268 ± 3034
Completeness (%)	89.2 ± 4.3	92.5 ± 3.9	96.7 ± 5.0	96.6 ± 3.5	96.9 ± 5.0	99.6 ± 0.4
*R*_work_ (%)	15.4 ± 1.6	14.6 ± 1.3	18.8 ± 1.6	15.7 ± 0.8	15.1 ± 1.1	14.4 ± 0.1
*R*_free_ (%)	19.7 ± 1.1	19.0 ± 2.4	21.7 ± 2.0	18.2 ± 1.2	17.8 ± 1.6	16.6 ± 0.4
R.m.s.d., bond lengths (Å)	0.028 ± 0.005	0.026 ± 0.005	0.022 ± 0.002	0.024 ± 0.002	0.015 ± 0.003	0.011 ± 0.001
R.m.s.d., angles (°)	2.25 ± 0.18	2.17 ± 0.20	2.11 ± 0.15	2.30 ± 0.12	1.56 ± 0.18	1.37 ± 0.03

**Table 2:** Ligands identified Sound pulses were used to harvest 96 proteinase K crystal aliquots onto micro-­meshes (25 nl) and then to combine them with 96 chemicals from a non­hazardous fragment mini-library (10 nl, 200 m*M* concentration). Of these 96 fragment-screening trials, 13 did not yield structures (including five that that failed to index correctly). X-ray diffraction was used to screen for binding in the remaining 83 structures and three chemicals were identified in the electron-density difference maps (80 were native). Values in parentheses are for the outer resolution shell.

	Bicine	ADA	Tartrate
Unit-cell parameters			
*a* = *b* (Å)	67.72	68.26	68.04
*c* (Å)	107.32	106.42	102.29
Resolution (Å)	1.71 (1.76–1.71)	1.63 (1.67–1.63)	1.50 (1.54–1.50)
*R*_merge_ (%)	17.5 (57.6)	16.5 (79.4)	7.6 (17.7)
CC_1/2_, outer shell	85.2	89.2	97.0
〈*I*/σ(*I*)〉	9.4 (1.8)	12.4 (2.5)	21.8 (2.4)
Multiplicity	12.6	12.7	10.2
Unique reflections	26111	30334	36472
Completeness (%)	99.8	99.7	97.9
*R*_work_ (%)	15.2 (34.9)	18.8 (32.5)	12.4 (21.3)
*R*_free_ (%)	17.3 (34.3)	24.9 (37.4)	15.7 (29.0)
R.m.s.d., bond lengths (Å)	0.024	0.020	0.025
R.m.s.d., angles (°)	2.10	1.84	2.21
Mean atomic *B* value (Å^2^)	15.47	23.84	8.69
No. of Ca atoms	2	1	1
Figure	Fig. 7 ▸(*a*)	Fig. 7 ▸(*b*)	Fig. 7 ▸(*c*)
PDB code	5whw	5wjh	5wjg

## Footnotes

1 Recently, a number of high-visibility applications for acoustic crystal handling at free-electron lasers have been described (Fuller *et al.*, 2017 ▸; Young *et al.*, 2016 ▸; Roessler *et al.*, 2016 ▸; Tsujino & Tomizaki, 2016 ▸; Boutet *et al.*, 2016 ▸). Similar devices could be adapted to inject crystals directly from an acoustically compatible crystallization plate (such as the MiTeGen *In Situ*-1 plate) and into the X-ray beam at synchrotron facilities.

2 Viola and coworkers conclude that ‘genuine demand exists in the high-throughput community as well as in pharmaceutical production pipelines, justifying the effort and resources to develop autonomous harvesting’.

3 Using *in situ* plates, X-ray data can be obtained without having to harvest the crystals, so readers may be confused as to why we selected them for this experiment. The thin and flat crystallization surface that promotes high-quality *in situ* data also facilitates acoustic crystal mounting. We experimented with many other kinds of crystallization plates that proved to be unsuitable for acoustic harvesting.

4 The mini-fragment library consisting of 96 nonhazardous chemicals was screened as part of a high-school-level structural biology course organized by the Office of Educational Programs at Brookhaven National Laboratory (BNL).

5 For manual screening, each crystal was transferred from the crystallization well to a solution containing mother liquor and 100 m*M* ligand. After 10 min cryoprotectant was added and the crystals were manually harvested onto cryo-loops for further diffraction measurements.
