# Supplementary material for: Using sound pulses to solve the crystal-harvesting bottleneck
Source: Acta Crystallogr D Struct Biol. 2018 Oct 2;74(Pt 10):986–99. doi: 10.1107/S2059798318011506 (PMC6173054; doi:10.1107/S2059798318011506)
Supplement: Supplementary file 1 [file d-74-00986-sup1.pdf]

S1 - Supplementary materials and methods: Detailed crystallization protocols

1. Thermolysin crystals were grown by equilibrating 330 mg/mL thermolysin (Sigma-Aldrich® T7902) in 1.45 M calcium chloride, 50 mM Tris pH 7.5, and 45% DMSO over a reservoir solution containing de-ionized water (crystals cryo-protected by slowly adding 30% ethylene glycol to the crystal containing drop).
2. Lysozyme crystals were grown by equilibrating 60 mg/mL lysozyme (Sigma-Aldrich® L4919) in 50 mM sodium acetate pH 4.6, 2% sodium chloride and 2.5% glycerol over a reservoir solution containing 8% sodium chloride and 10% glycerol (no additional cryo-protectant was needed).
3. Proteinase K crystals were grown by equilibrating 25 mg/mL proteinase K (Worthington Biochemical® LS004224) in 40 mM calcium chloride, 400 mM sodium nitrate, and 50 mM BisTris (pH 6.5) over a reservoir containing 160 mM calcium chloride, 1.6 M sodium nitrate, and 200 mM BisTris (pH 6.5) (crystals cryo-protected by addition of 30% ethylene glycol).
4. Trypsin crystals were grown by equilibrating 15 mg/mL trypsin (Sigma-Aldrich® T1426) plus 5 mg/mL benzamidine and 10 mM calcium chloride in 20 mM HEPES (pH 7.0) with 3.75% PEG 3350 and 5% glycerol over a reservoir containing 15% PEG 3350 and 20% glycerol (no additional cryo-protection needed).
5. Ferritin crystals were grown by depositing a single 10 uL aliquot of hot agarose containing 100 mM $CdSO_4$ on one side of the crystallization shelf, and placing into contact with it 5 uL of ferritin (Sigma-Aldrich® F4503) and 5 uL of 1.6 M $AmSO_4$, 0.2 M Tris (pH 7.7).

S2 - Supplementary materials and methods: Detailed description of plate modifications

A. Fabricating a polypro assembly: Five commonly used crystallization plates were cut into pieces that were one crystallization chamber wide and five crystallization chambers long ("plate fragments" hereafter) (Figure 2A, inset). Each of the five plate fragments was transferred to an acoustically transparent plate that was modified to couple the non-acoustic lab-ware to our acoustic system. The middle section of a 384 well polypropylene microplate™ (Labcyte Inc) was modified by grinding down the honeycomb structure to a total height of 1.7 mm. The top of the modified polypropylene plate was coated with ~ 3 mm thick agarose pillow to acoustically couple the MiTeGen plate fragment with the crystal containing drops to the Echo 550. Plate fragments (from non-acoustic lab-ware) were deposited onto the agarose pillow, and this completed the structure of the hybrid plate ("polypro assembly" hereafter) (Figure 2A). Care was taken to avoid any air gap between the plastic and the agarose pillow. The apparatus and specimens were photographically documented at each stage of the crystal transfer procedure.

B. Fabricating a MiTeGen assembly: An entire 384 well polypropylene microplate™ (Labcyte Inc) was modified by grinding down the honeycomb structure to a total height of 1.7 mm (Figure 2B, inset). The ground-down polypro was coated with a 3 mm thick agarose pillow (see paragraph above) and coupled to the bottom of a MiTeGen plate before the agarose cooled. Before the agarose cooled, the position of the ground-down polypro was adjusted so that each well had the same position relative to the edge of the MiTeGen plate as it would have to the edge of an intact polypro plate. This hybrid plate ("MiTeGen assembly" hereafter) was used to acoustically couple the entire MiTeGen plate to the Echo 550. The MiTeGen assembly was used to grow protein crystals, and then to harvest those crystals onto micro-meshes for X-ray data collection (§2.5). The apparatus and specimens were photographically documented at each stage of the crystal transfer procedure.

S3 - Supplementary results: Testing results from many plates using the polypro assembly

With some effort it was possible to couple all of the plate fragments to the polypropylene plate as described in §2.1.1. In some cases, acoustic harvesting was not possible because too much acoustic energy was lost (see §3.1) so that the momentum transferred to the crystal slurry was insufficient to eject a droplet. However, MiTeGen plates and CrystalDirect plates did not greatly diminish the acoustic signal.

<u>Diffraction from acoustically harvested thermolysin crystals is comparable to controls</u>: Slightly better data were observed from crystals that were manually harvested onto *matching sized cryo-loops* compared to thermolysin crystals that were acoustically harvested onto micro-meshes (S10 Table 1). More solvent was observed around crystals on micro-meshes (both acoustically harvested and hand harvested) compared to crystals that were carefully hand-harvested onto matching sized cryo-loops. The additional background from this solvent and the mesh possibly accounts for the difference in data quality. The mean resolution limit for the 10 acoustically harvested thermolysin crystals at I/σI = 1.0 was 2.06 Å ± 0.32 Å (R_{merge} = 13.5% ± 4.0%) compared to 1.73 Å ± 0.07 Å (R_{merge} = 7.7% ± 1.7%) for manually harvested crystals (S11). However, no significant difference was observed between lysozyme crystals that were acoustically harvested onto micro-meshes compared to crystals that were hand harvested onto micro-meshes (acoustic harvested: mean resolution 1.85 Å ± 0.30 Å, R_{merge} 12.9% ± 3.2%; manually harvested: mean resolution 1.89 Å ± 0.23 Å, R_{merge} 11.5% ± 2.8%; S15).

S4 - Supplementary results: Testing results from MiTeGen assembly

The X-ray data that were obtained from eight acoustically harvested lysozyme crystals were comparable with the data collected from the eight control lysozyme crystals. As was the case with crystals harvested from a polypro assembly (§3.2), when comparing acoustic harvesting onto micro-meshes to hand harvesting onto cryo-loops, the R_{merge} was slightly lower for the hand mounted crystals (Table 1). The mean resolution limit (I/σI = 1.0) was 1.41 Å ± 0.10 Å (R_{merge} = 11.2% ± 3.2%) compared to 1.40 Å ± 0.01 Å (R_{merge} = 7.4% ± 1.4%) (data in S13-supplementary table 4).

Supplementary figure S1: Image of plate inside of Echo.

An image of the acoustically compatible plate within the Echo instrument, used to transfer crystals onto a pin platform (a). The pedestal of the MiTeGen in *situ-1* plate (b) must be abraded to bring the specimen platform (arrows) close enough to the transducer so that acoustic pulses can reach the crystals inside the crystallization plate.

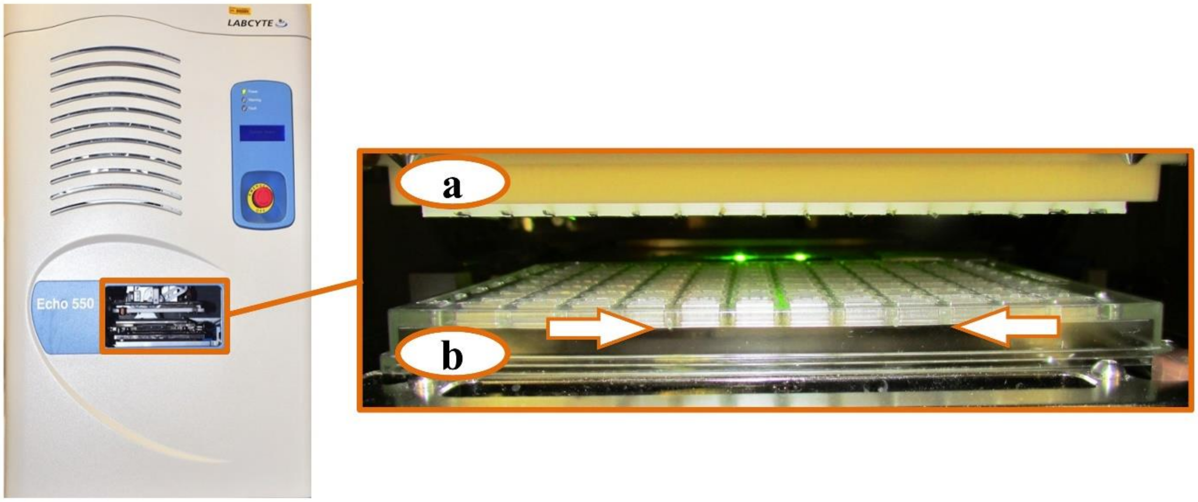

Supplementary figure S2: Growing crystals in a Bingham fluid.

To facilitate the harvesting of large crystals (> 50 µm) the mother liquor was made into a Bingham fluid by adding 0.15% agarose (lysozyme and proteinase K require the same concentration of agarose to form a Bingham fluid). A Bingham fluid can be acoustically ejected like a liquid but prevents large crystals from settling to the bottom of the crystallization well. To visualize the 3D appearance of our Bingham suspensions, the microscope described in §2.2 was used to obtain a stack of 10 bright-field images from the crystallization well. Each image in the bright field stack had a different focus, such that the stack evenly partitioned the 3.2 mm well depth. The bright field stack was converted into a 3D visualization by custom NSLS II software (Gofron et al., 2017). 10 nL of crystallization fluid was then acoustically harvested and the number of ejected crystals was counted. A new bright-field stack was then obtained, and this process was repeated ten times. By examining the ten 3D models, it was possible to determine the location from which each acoustically harvested crystal was ejected. This demonstrated that the ejected crystals were harvested from the surface layer (see Figure 9). Hence, crystals near the surface layer tended to move towards the ejection point, while deeper crystals remained stationary. The two focus stacked images below show (i) that one crystal (red box) was acoustically harvested, (ii) that nearby crystals near the surface (green arrows and circles) moved towards the ejection point, and (iii) that nearby crystals near the bottom of the plate (blue circles) remained stationary.

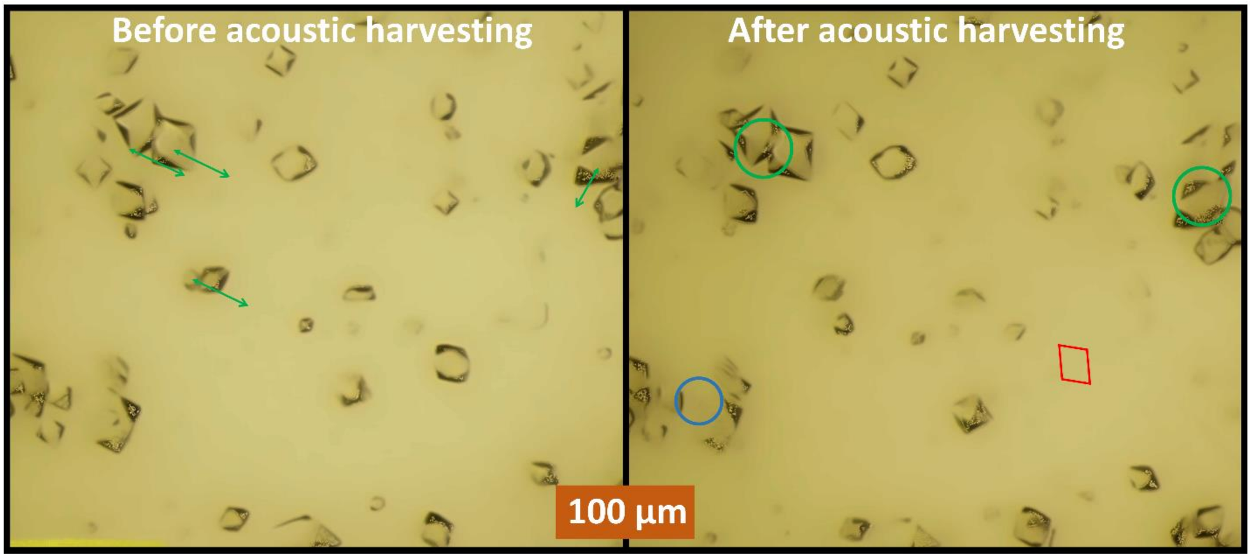

Supplementary figure S3: Instructions for preparing acoustically compatible MiTeGen plates.

The 1.22 mm edge pedestal must be abraded before crystals can be acoustically harvested from a MiTeGen *in situ-1™* crystallization plate. The 15 minute dry abrasion procedure was performed using coarse 100 grit sand paper (10 minutes), followed by wet abrasion using 320 grit sandpaper to make sure that the vertical component of the edge pedestal was completely removed (5 minutes). The horizontal section of the edge pedestal of the modified plate should be smooth to the touch, with no remnant of the vertical component. It is possible for the plate to become warped if too much force is applied during the abrasion procedure. A slightly warped plate can still be used for acoustic harvesting, provided slight downward pressure is applied to keep the plate flat while it is loaded into the ECHO 550.

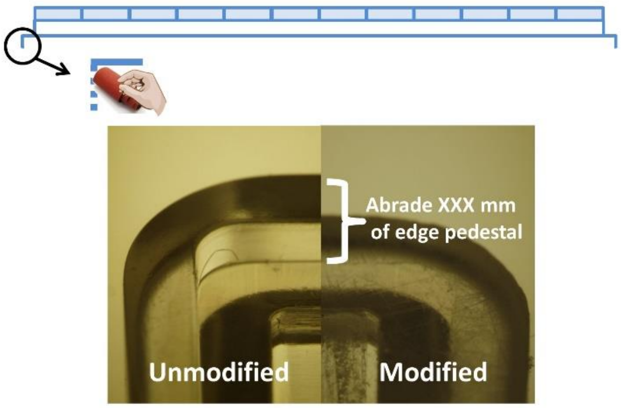

Supplementary figure S4: Two known ligands acoustically added to lysozyme.

ADE was used to separately combine lysozyme crystals with 33 chemicals from a chemical minilibrary, including two known lysozyme ligands (benzamidine and NAG). Panel A shows the difference omit map for the benzamidine ligand bound to lysozyme, and panel B shows the difference omit map the NAG ligand bound to lysozyme (both maps were contoured at 3σ). No other chemicals in the mini-library were observed to bind to lysozyme.

Note that the same technique was used to screen a 304 fragment library for binding to trypsin crystals (data for these experiments are not shown). Trypsin is a model protein for fragment screening (Newman et al., 2009). The fragment library consisted of 45 cocktails, each of which had between six and ten distinct chemicals solvated in 20 mM HEPES (pH 7.5), 0.15 M sodium chloride, 10% glycerol, and 5 mM DTT (dithiothreitol). 25 nL of trypsin crystal slurry was ejected onto each of 45 micro-meshes and combined with 10 nL of each cocktail (the chemicals were not allowed to dry because some of the chemicals rapidly degrade if desiccated). The trypsin crystals had a rod-shaped habit approximately 50 µm long. Less than one minute was required to harvest 45 crystal-containing droplets from a slurry of trypsin crystals (in a single well of a MiTeGen crystallization plate) onto 45 distinct micro-meshes, and to combine each crystal with one of the 45 chemical cocktails. However, though diffraction data were obtained from each of the trypsin crystals, none of the 304 chemicals in the cocktail library were observed to bind to trypsin. This may be because the chemicals in the cocktail libraries were present at lower concentration than is possible with individually solvated chemicals (hence low affinity interactions are difficult to detect).

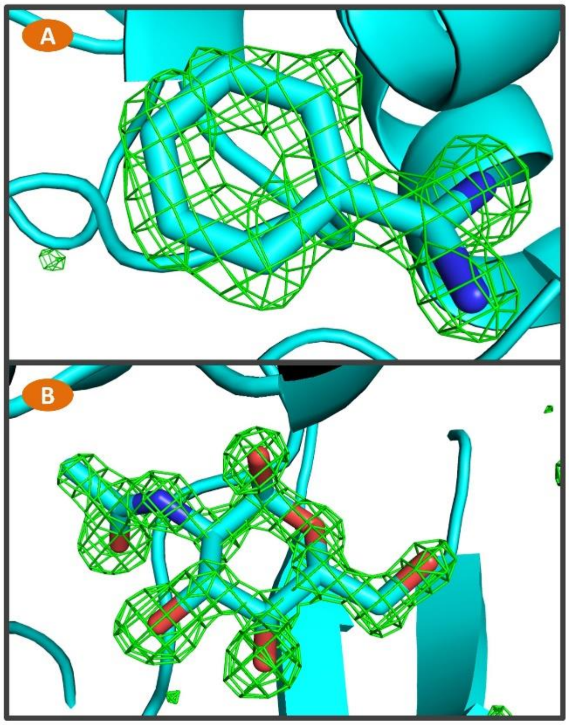

Supplementary figure S5: Echo 550 settings for crystal harvesting from each location.

There are three locations from which it was attempted to acoustically harvest crystals within each crystallization well of a MiTeGen InSitu-1 plate (pictured). For each crystallization well, the matching settings for the Echo 550 are shown (using the 1536 well LDV source template). For example, if an experimenter wishes to harvest crystals from the center location on well A2 of a MiTeGen plate, the experimenter should select “B6” as the source location on the 1536 well LDV template. Positions shown with red letters were not reliable for acoustic crystal harvesting.

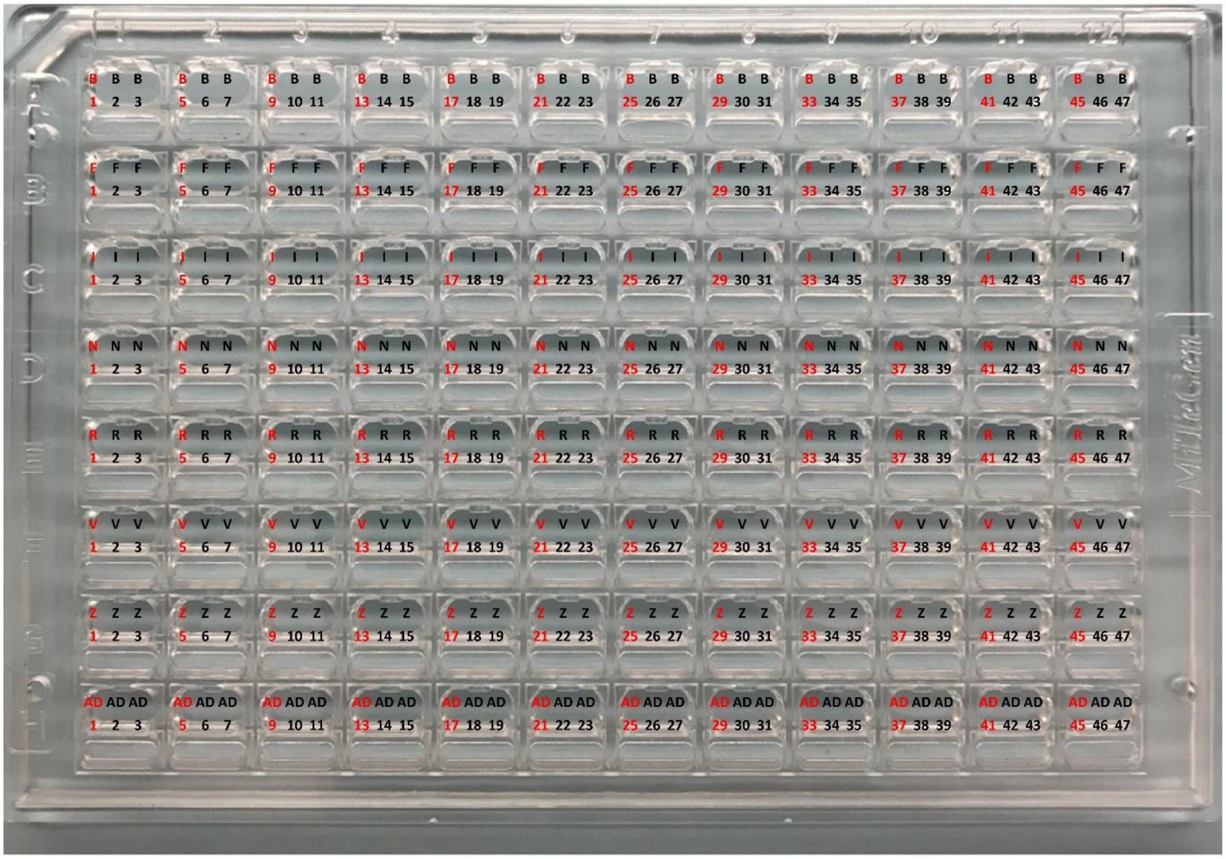

Supplementary Table S1: Comparison of time for manual and acoustic fragment soaking, crystal harvesting, and cryo-cooling

The differences between the per-fragment times for manual versus acoustic specimen preparation are shown. Two cases are illustrated, to account for situations where cryo-protectant must be separately added prior to crystal harvesting (right; 350 seconds per fragment) and when no cryo-protectant is needed (left; 224 seconds per fragment). The manual specimen preparation times were observed during two trials performed by a well-trained but inexperienced research assistant. A skilled technician likely would achieve 50% faster specimen preparation (~3 minutes per fragment with cryo-protectant, ~2 minutes per fragment with no cryo-protectant). By comparison, an inexperienced operator was able to acoustically prepare one specimen every 1 minutes (adding cryo-protectant has negligible impact).

Note that the Echo 550 cannot be used to transfer the micro-meshes (containing fragment screening experiments) into a cryo-cooled puck. This manual pin-transfer remains the slowest step in acoustic specimen preparation. However, this step can be automated using a six-axis robot (ttps://www.youtube.com/watch?v=Q6HTA1wRZyQ). Including robotic pin-transfer, the time required per screened fragment from ~60 seconds to ~15 seconds.

	No cryo-protectant			With cryo-protectant		
	Manual	Acoustic (manual cryo-cooling)	Acoustic (robotic cryo-cooling)	Manual	Acoustic (manual cryo-cooling)	Acoustic (robotic cryo-cooling)
Combine crystal + chemical (s)	58.4	0.5	0.5	52.2	0.5	0.5
Add cryo-protectant (s)	0.0	0.5	0.5	55.8	1.0	1.0
Harvest crystal and freeze (s)	165.6	56.3	13.0	242.2	56.3	13.0
Total time per screen (S)	224.0	57.3	14.0	350.2	57.8	14.5

Supplementary Table S2: Echo 550 settings for crystal harvesting.

The same information that is shown graphically on S9 is tabulated here. For example, an investigator who wishes to harvest crystals from the middle position of well A2 in a MiTeGen plate should use the B6 setting on the 1536 LDV template. Positions shown in red are not reliable for acoustic harvesting, because the acoustic harvesting pulses cannot propagate very close to the edge of the crystallization wells. Note that the authors are working with the Echo 550 vendor to generate simple to use software for accessing desired source plate locations in a flexible way.

	1			2			3			4			5			6		
A	B1	B2	B3	B5	B6	B7	B9	B10	B11	B13	B14	B15	B17	B18	B19	B21	B22	B23
B	F1	F2	F3	F5	F6	F7	F9	F10	F11	F13	F14	F15	F17	F18	F19	F21	F22	F23
C	I1	I2	I3	I5	I6	I7	I9	I10	I11	I13	I14	I15	I17	I18	I19	I21	I22	I23
D	N1	N2	N3	N5	N6	N7	N9	N10	N11	N13	N14	N15	N17	N18	N19	N21	N22	N23
E	R1	R2	R3	R5	R6	R7	R9	R10	R11	R13	R14	R15	R17	R18	R19	R21	R22	R23
F	V1	V2	V3	V5	V6	V7	V9	V10	V11	V13	V14	V15	V17	V18	V19	V21	V22	V23
G	Z1	Z2	Z3	Z5	Z6	Z7	Z9	Z10	Z11	Z13	Z14	Z15	Z17	Z18	Z19	Z21	Z22	Z23
H	AD1	AD2	AD3	AD5	AD6	AD7	AD9	AD10	AD11	AD13	AD14	AD15	AD17	AD18	AD19	AD21	AD22	AD23

	7			8			9			10			11			12		
A	B25	B26	B27	B29	B30	B31	B33	B34	B35	B37	B38	B39	B41	B42	B43	B45	B46	B47
B	F25	F26	F27	F29	F30	F31	F33	F34	F35	F37	F38	F39	F41	F42	F43	F45	F46	F47
C	I25	I26	I27	I29	I30	I31	I33	I34	I35	I37	I38	I39	I41	I42	I43	I45	I46	I47
D	N25	N26	N27	N29	N30	N31	N33	N34	N35	N37	N38	N39	N41	N42	N43	N45	N46	N47
E	R25	R26	R27	R29	R30	R31	R33	R34	R35	R37	R38	R39	R41	R42	R43	R45	R46	R47
F	V25	V26	V27	V29	V30	V31	V33	V34	V35	V37	V38	V39	V41	V42	V43	V45	V46	V47
G	Z25	Z26	Z27	Z29	Z30	Z31	Z33	Z34	Z35	Z37	Z38	Z39	Z41	Z42	Z43	Z45	Z46	Z47
H	AD25	AD26	AD27	AD29	AD30	AD31	AD33	AD34	AD35	AD37	AD38	AD39	AD41	AD42	AD43	AD45	AD46	AD47

Supplementary Table S3: Statistics for thermolysin crystals harvested from polypro assembly.

The top table shows refinement statistics for thermolysin crystals acoustically harvested onto micro-meshes, and the bottom table shows refinement statistics for similar crystals manually harvested onto loops. Refls = unique reflections, RMS BL = root mean square difference between observed and ideal bond lengths (in Å), RMS Ang = root mean square difference between observed angles and ideal angles (in degrees).

			Acoustically harvested crystals					
	Res	Refls(Å)	Compl (%)	R_{merge}(%)	R_{work}(%)	R_{free}(%)	RMS BL	RMS Ang
1	2.58	26614	99.9	20.2	17.2	21.5	0.021	1.94
2	2.58	22266	84.0	19.2	14.7	18.9	0.018	1.79
3	2.45	24383	92.2	17.2	14.4	18.5	0.017	1.72
4	1.74	37014	100.0	7.9	14.0	15.8	0.012	1.40
5	1.72	44241	99.8	10.9	14.6	17.0	0.011	1.38
6	1.94	37222	99.8	12.7	15.1	17.1	0.014	1.50
7	1.97	42294	95.3	12.7	16.1	17.4	0.014	1.52
8	1.84	39780	99.7	12.4	14.7	16.7	0.012	1.41
9	1.99	26915	99.9	13.4	13.8	16.5	0.013	1.49
10	1.83	37076	99.0	8.6	16.6	19.1	0.013	1.48
Average	2.06	33781	96.9	13.5	15.1	17.8	0.015	1.56
StDev	0.32	7553	5.0	3.9	1.1	1.6	0.003	0.18

			Hand harvested controls					
	Res	Refls(Å)	Compl (%)	R_{merge}(%)	R_{work}(%)	R_{free}(%)	RMS BL	RMS Ang
1	1.83	36753	99.5	7.5	14.3	16.2	0.011	1.41
2	1.88	36584	98.8	8.7	14.2	16.2	0.012	1.43
3	1.72	44377	99.9	8.5	14.4	17.0	0.011	1.36
4	1.79	39742	99.9	11.2	14.3	16.1	0.010	1.35
5	1.70	39421	99.2	7.3	14.4	16.2	0.010	1.35
6	1.64	44240	100.0	5.0	14.4	16.9	0.010	1.35
7	1.70	39547	100.0	5.3	14.3	16.9	0.011	1.36
8	1.72	43895	99.6	8.4	14.6	17.3	0.011	1.38
9	1.64	44031	99.9	6.5	14.3	16.8	0.011	1.36
10	1.72	44094	99.7	8.5	14.4	16.9	0.011	1.36
Average	1.73	41268	99.6	7.7	14.4	16.6	0.011	1.37
StDev	0.07	3034	0.4	1.7	0.1	0.4	0.001	0.03

Supplementary Table S4: Statistics for lysozyme crystals harvested from MiTeGen assembly

The top table shows refinement statistics for lysozyme crystals acoustically harvested onto micro-meshes, and the bottom table shows refinement statistics for similar crystals manually harvested onto loops. Refls = unique reflections, RMS BL = root mean square difference between observed and ideal bond lengths (in Å), RMS Ang = root mean square difference between observed angles and ideal angles (in degrees), and the last shell Δλ=0.04Å. Data were obtained at CHESS for four acoustically harvested lysozyme crystals and four controls. Additional data were obtained at SSRL for four acoustically harvested lysozyme crystals and four controls. Where possible, data were processed to $I/\sigma I^{last} \approx 4.0$ (hand harvested controls #4 and #5 have a higher $I/\sigma I^{last}$ due to the minimum achievable detector distance).

Acoustically harvested crystals															
	Res(Å)	a=b (Å)	c (Å)	Refls	Compl(%)	R_{mer}(%)	R_{mer}^{last}(%)	$CC_{1/2}^{last}$	I/σI	$I/\sigma I^{last}$	R_{work}(%)	R_{free}(%)	BL	Ang	Mult
1	1.36	78.68	37.21	25663	98.0	7.9	56.1	0.917	56.8	4.0	19.2	22.5	0.023	2.20	12.5
2	1.55	78.66	37.14	17543	99.4	10.1	54.8	0.947	41.8	6.2	19.3	21.9	0.020	1.98	9.1
3	1.42	78.87	37.22	20077	83.9	9.1	51.2	0.928	63.4	3.8	18.2	20.9	0.023	2.16	21.9
4	1.61	78.97	37.42	15614	97.2	18.5	65.9	0.826	22.3	4.3	22.1	25.2	0.020	1.99	8.0
5	1.42	78.66	37.18	22881	99.7	8.5	61.2	0.939	52.2	4.8	17.9	19.6	0.025	2.29	13.0
6	1.54	78.58	37.16	17598	96.3	14.5	80.2	0.891	35.2	3.7	19.9	21.4	0.023	2.16	11.6
7	1.46	78.65	37.11	20846	100.0	9.3	69.5	0.930	34.4	3.8	16.2	18.3	0.024	2.25	14.0
8	1.71	77.67	37.12	12859	99.5	13.1	74.7	0.911	39.1	3.9	17.8	23.5	0.019	1.84	12.3
Average	1.51	78.59	37.20	19135	96.7	11.4	64.2	0.911	43.2	4.3	18.8	21.7	0.022	2.11	12.8
StDev	0.11	0.37	0.09	3827	5.0	3.4	9.5	0.036	12.6	0.8	1.6	2.0	0.002	0.15	3.9

Hand harvested controls															
	Res(Å)	a=b (Å)	c (Å)	Refls	Compl(%)	R_{mer}(%)	R_{mer}^{last}(%)	$CC_{1/2}^{last}$	I/σI	$I/\sigma I^{last}$	R_{work}(%)	R_{free}(%)	BL	Ang	Mult
1	1.40	78.10	36.86	21591	98.0	5.4	71.7	0.845	94.5	3.8	16.3	18.6	0.023	2.25	18.6
2	1.41	77.97	36.96	21976	99.6	8.1	96.4	0.810	84.3	4.2	16.1	20.0	0.025	2.30	18.7
3	1.59	78.17	36.95	16090	88.4	7.5	84.2	0.871	84.3	4.2	16.9	20.0	0.021	2.04	15.5
4	1.40	78.71	36.95	21897	97.9	5.8	35.2	0.963	97.3	11.8	15.0	17.3	0.024	2.26	19.8
5	1.40	78.38	36.91	21306	95.9	6.1	32.0	0.965	91.5	13.0	16.6	18.2	0.025	2.30	18.0
6	1.40	78.75	36.91	22082	98.9	6.4	65.3	0.882	92.7	4.3	15.1	17.0	0.026	2.40	19.5
7	1.40	78.58	36.92	21139	94.8	6.2	64.3	0.899	94.6	5.9	14.9	18.3	0.025	2.38	18.2
8	1.40	78.69	36.92	22177	99.2	6.1	42.8	0.842	87.0	3.6	15.0	16.5	0.026	2.45	14.9
Average	1.43	78.42	36.92	21032	96.6	6.5	61.5	0.885	90.8	6.3	15.7	18.2	0.024	2.30	17.9
StDev	0.07	0.29	0.03	1900	3.5	0.8	21.7	0.052	4.7	3.5	0.8	1.2	0.002	0.12	1.7

Supplementary Table S5: Statistics for proteinase K harvested from MiTeGen plate.

The top table shows refinement statistics for proteinase K crystals acoustically harvested onto micro-meshes, and the bottom table shows refinement statistics for similar crystals manually harvested onto micro-meshes. Refls = unique reflections, RMS BL = root mean square difference between observed and ideal bond lengths (in Å), RMS Ang = root mean square difference between observed angles and ideal angles (in degrees), and the last shell Δλ=0.06Å. The data from manually harvested crystals were merged, and the data from acoustically harvested crystals were separately merged. The R_{work} and R_{free} values were similar, indicating that there was no significant difference in isomorphism within each category (acoustic R_{work}=15.0%, R_{free}=17.7%; manual R_{work}=15.2%, R_{free}=18.1%).

Acoustically harvested crystals															
	Res	a=b (Å)	c (Å)	Refls	Compl(%)	R_{mer}(%)	R_{mer}^{last}(%)	$CC_{1/2}^{last}$	I/σI	I/σI ^{last}	R_{work}(%)	R_{free}(%)	BL	Ang	Mult
1	1.65	67.83	107.66	25044	91.1	8.5	62.9	0.719	15.5	1.9	13.7	17.9	0.030	2.34	8.3
2	1.44	67.64	107.55	34114	82.1	9.5	75.8	0.705	16.3	1.0	17.7	19.8	0.033	2.28	6.9
3	2.17	68.11	106.73	11590	93.4	13.8	73.9	0.868	11.5	2.5	14.0	21.0	0.020	1.95	8.5
4	1.60	67.85	107.67	26986	90.3	12.2	68.1	0.820	14.4	1.9	16.1	20.0	0.028	2.44	7.8
Average	1.72	67.86	107.40	24434	89.2	11.0	70.2	0.778	14.4	1.8	15.4	19.7	0.028	2.25	7.9
StDev	0.27	0.17	0.39	8148	4.3	2.1	5.1	0.068	1.8	0.5	1.6	1.1	0.005	0.18	0.6

Hand harvested controls															
	Res	a=b (Å)	c (Å)	Refls	Compl(%)	R_{mer}(%)	R_{mer}^{last}(%)	$CC_{1/2}^{last}$	I/σI	I/σI ^{last}	R_{work}(%)	R_{free}(%)	BL	Ang	Mult
1	1.97	68.07	108.05	14718	90.5	13.3	73.3	0.672	11.1	1.9	14.7	20.7	0.022	2.09	7.3
2	1.95	67.84	102.37	16757	99.1	15.1	80.3	0.871	9.5	2.2	16.7	22.0	0.019	1.90	6.4
3	1.72	67.71	107.43	22263	91.0	12.1	63.4	0.821	12.1	2.3	13.7	16.5	0.028	2.23	8.6
4	1.57	67.83	107.56	28584	89.3	7.0	56.9	0.739	14.5	1.7	13.3	16.7	0.033	2.44	5.8
Average	1.80	67.86	106.35	20581	92.5	11.9	68.5	0.776	11.8	2.0	14.6	19.0	0.026	2.17	7.0
StDev	0.17	0.13	2.31	5382	3.9	3.0	9.0	0.076	1.8	0.2	1.3	2.4	0.005	0.20	1.1

Supplementary Table S6: Statistics for lysozyme harvested from polypro assembly.

The top table shows refinement statistics for lysozyme crystals acoustically harvested onto micro-meshes, and the bottom table shows refinement statistics for similar crystals manually harvested onto micro-meshes. Refls = unique reflections, RMS BL = root mean square difference between observed and ideal bond lengths (in Å), RMS Ang = root mean square difference between observed angles and ideal angles (in degrees).

Acoustically harvested crystals								
	Res	Refls	Compl (%)	R_{merge}(%)	R_{work}(%)	R_{free}(%)	RMS BL	RMS Ang
1	1.49	29233	99.9	8.1	16.7	18.4	0.007	1.22
2	1.85	19128	98.9	13.8	16.7	19.4	0.010	1.31
3	1.80	22140	92.4	13.5	17.7	20.0	0.011	1.32
4	1.91	17400	99.3	15.2	17.1	19.0	0.011	1.37
5	1.72	21292	99.6	12.9	17.1	18.8	0.009	1.28
6	2.10	13346	99.2	15.9	19.8	22.9	0.024	2.17
7	1.88	13416	99.8	13.5	16.7	19.7	0.011	1.34
8	2.58	9669	99.6	18.2	16.1	20.8	0.017	1.59
9	1.54	28040	96.7	8.2	17.3	18.9	0.008	1.25
10	1.61	21122	99.5	9.5	17.2	19.2	0.009	1.27
Average	1.85	19479	98.5	12.9	17.2	19.7	0.012	1.41
StDev	0.30	5971	2.2	3.2	1.0	1.3	0.005	0.27

Hand harvested controls								
	Res	Refls	Compl (%)	R_{merge}(%)	R_{work}(%)	R_{free}(%)	RMS BL	RMS Ang
1	1.91	17082	97.9	10.8	16.5	17.7	0.010	1.36
2	1.72	19216	99.8	9.6	16.7	18.9	0.009	1.29
3	1.66	23194	98.8	8.9	17.0	18.7	0.008	1.31
4	1.72	19174	99.9	8.4	16.4	18.5	0.009	1.28
5	1.91	17265	98.7	13.0	17.2	18.7	0.010	1.32
6	1.73	22646	95.9	10.0	17.7	19.7	0.009	1.29
7	2.32	10508	93.8	15.4	16.2	21.3	0.017	1.58
8	1.90	15897	99.5	13.5	17.5	19.2	0.013	1.49
9	2.31	12790	95.7	16.6	19.0	23.1	0.019	1.79
10	1.74	24426	93.4	9.1	17.1	19.6	0.008	1.26
Average	1.89	18220	97.3	11.5	17.1	19.6	0.011	1.40
StDev	0.23	4258	2.3	2.8	0.8	1.5	0.004	0.16
